# Supplementary material for: WRNIP1 Is Recruited to DNA Interstrand Crosslinks and Promotes Repair
Source: Cell Rep. 2020 Jul 7;32(1):107850. doi: 10.1016/j.celrep.2020.107850 (PMC7351111; doi:10.1016/j.celrep.2020.107850)
Supplement: Document S1. Figures S1–S7 [file mmc1.pdf]

Cell Reports, Volume *32*

Supplemental Information

WRNIP1 Is Recruited to DNA Interstrand Crosslinks and Promotes Repair

Anna Socha, Di Yang, Alicja Bulsiewicz, Kelvin Yaprianto, Marian Kupculak, Chih-Chao Liang, Andreas Hadjicharalambous, Ronghu Wu, Steven P. Gygi, and Martin A. Cohn

Figure S1

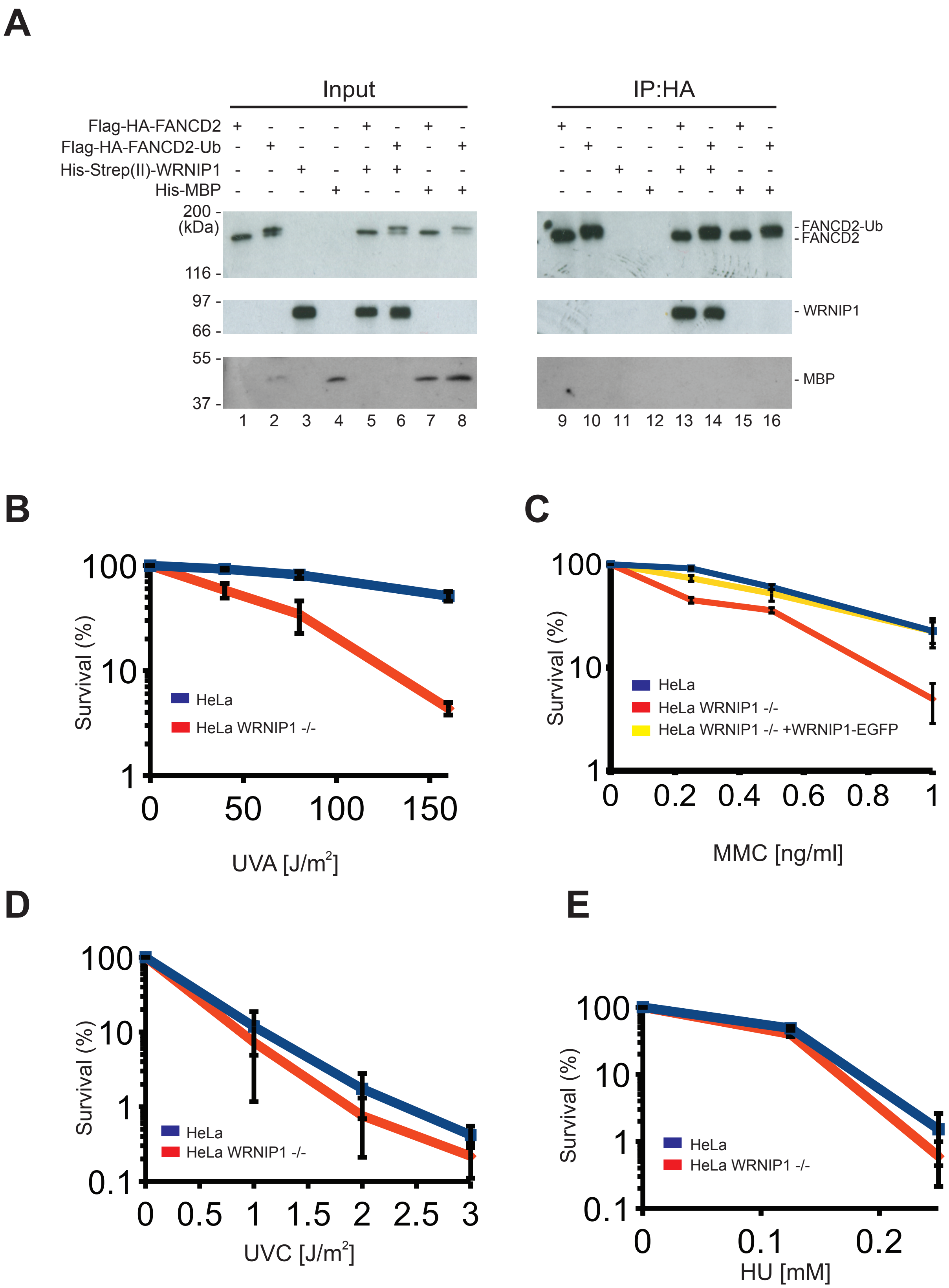

Figure S1. WRNIP1 is important for ICL repair. Relating to Figures 1 and 2. A) Immunoblot analysis of *in vitro* protein-protein interaction assay showing a direct interaction between WRNIP1 and FANCD2. B) The CRISPR/Cas9 technique was used to disrupt the *WRNIP1* gene in HeLa cells, creating a WRNIP1 deficient HeLa cell line. Clonogenic survival assay of HeLa and HeLa WRNIP1-/- cells in response to TMP/UVA (mean ± SEM, n=2). C) Clonogenic survival assay of HeLa and HeLa WRNIP1-/- cells in response to MMC (mean ± SEM, n=2). D) Clonogenic survival assay of HeLa and HeLa WRNIP1-/- cells in response to UVC (mean ± SEM, n=2). E) Clonogenic survival assay of HeLa and HeLa WRNIP1-/- cells in response to HU (mean ± SEM, n=2).

Figure S2

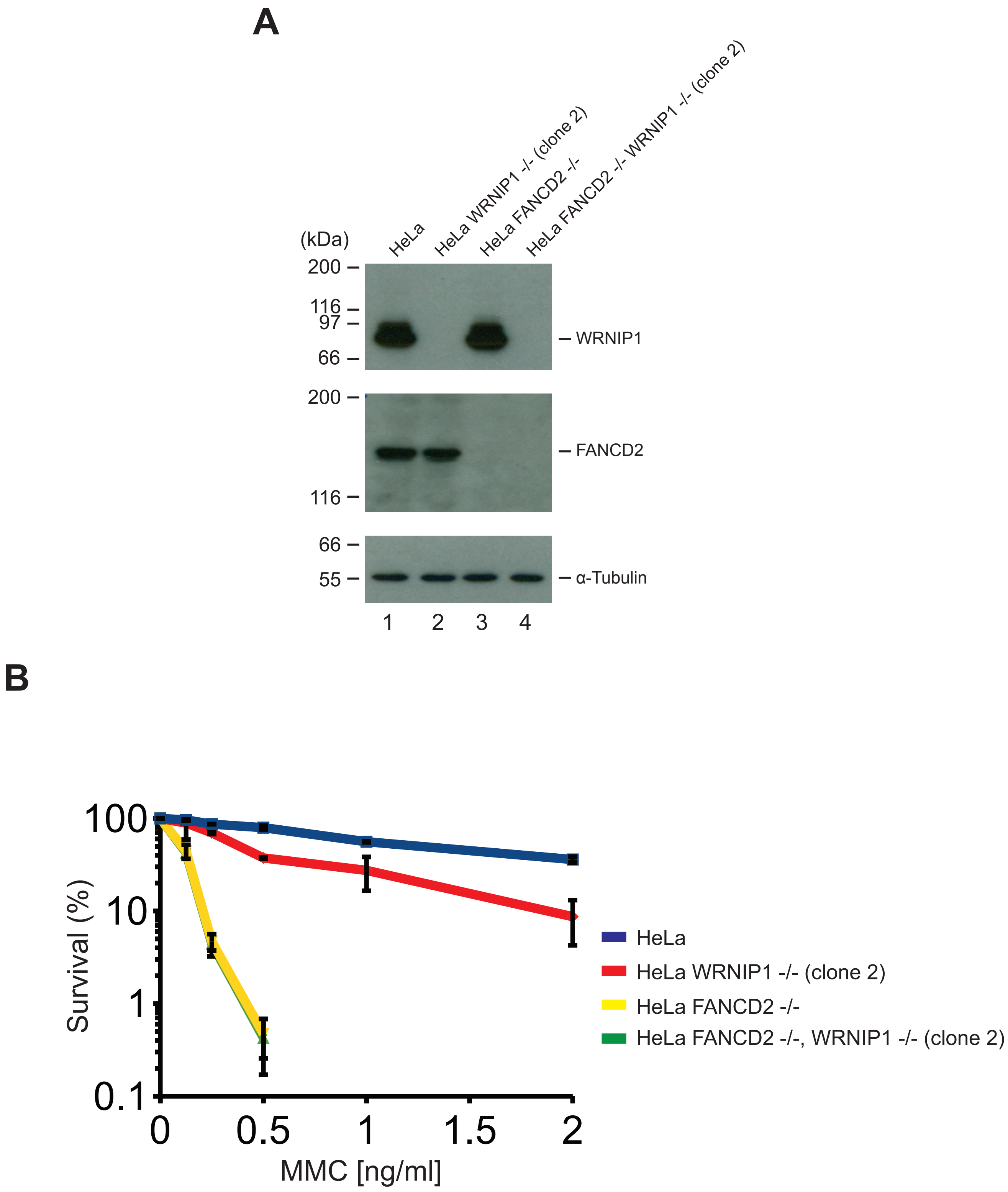

Figure S2. WRNIP1 is important for ICL repair. Relating to Figure 2. A) The CRISPR/Cas9 technique was used to disrupt the *WRNIP1* and *FANCD2* genes in HeLa cells, creating HeLa WRNIP -/- (clone 2), HeLa FANCD2 -/- and HeLa WRNIP -/- FANCD2 -/- (clone 2) cell lines. Immunoblot analysis. B) Clonogenic survival assay of the indicated cell lines in response to MMC. Mean ± SEM, n=2.

Figure S3

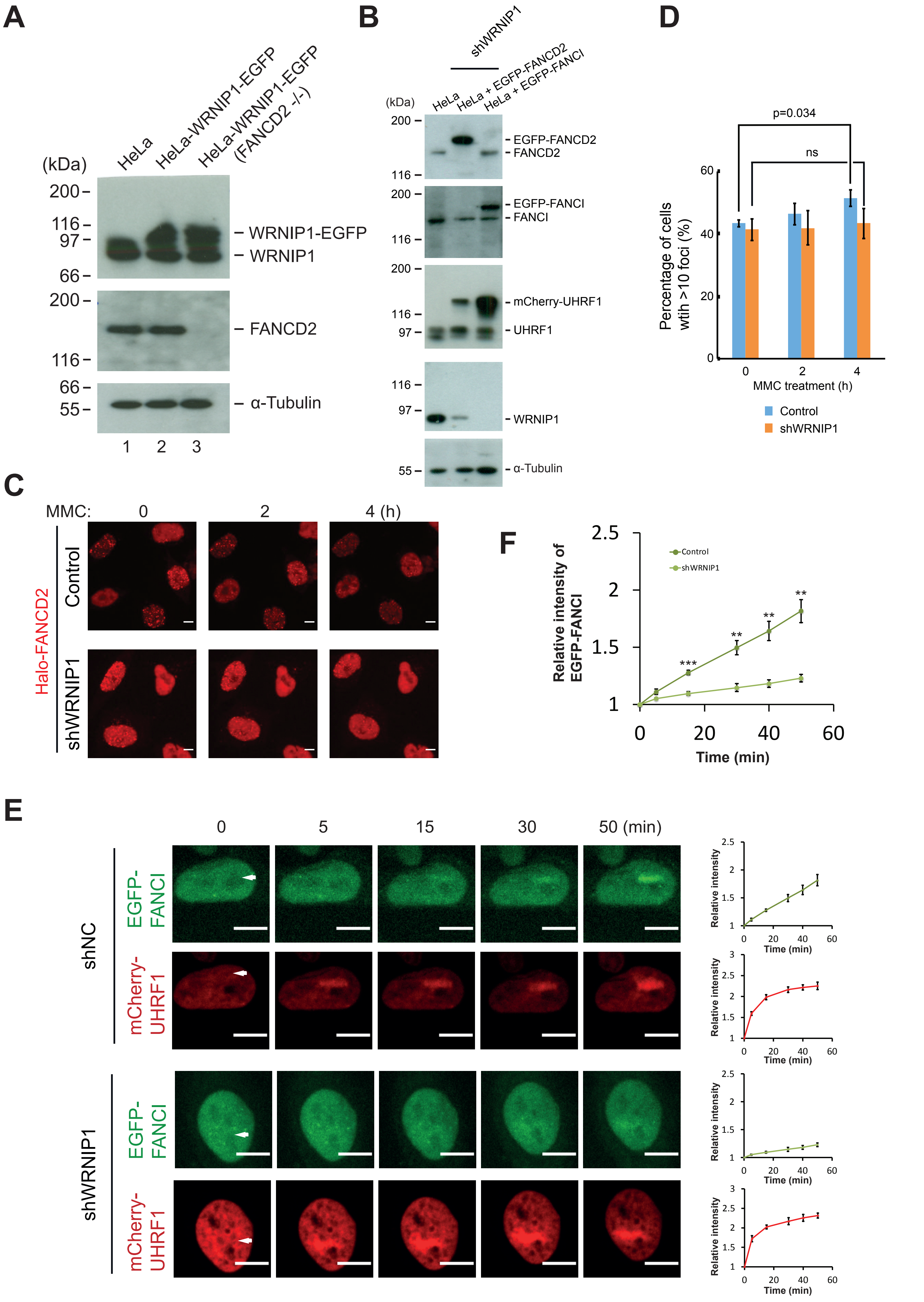

(legend on next page)

Figure S3. WRNIP1 is recruited to ICLs. Relating to Figure 3. A) Immunoblot analysis showing expression of WRNIP1-EGFP in Flp-in T-REx HeLa and Flp-in T-REx HeLa FANCD2 -/- cells. B) Immunoblot analysis showing expression of EGPF-FANCD2, EGPF-FANCI and mCherry-UHRF1 in HeLa cells expressing either a control shRNA encoding a scramble sequence, or, an shRNA targeting WRNIP1. C) Live-cell imaging of HeLa-Halo-FANCD2 cells and HeLa-Halo-FANCD2 cells where WRNIP1 was depleted using shRNA. Cells were treated with 160 ng/ml MMC and followed over time. Scale bar, 10 µm. D) Quantification of (C). A total of 373, 414 and 361 cells were quantified for control cells at 0, 2, and 4h time points, respectively. A total of 465, 588 and 511 cells were quantified for cells where WRNIP1 was depleted at 0, 2, and 4h time points, respectively. Mean ± SEM, ns, not significant. E) Live-cell imaging of HeLa-EGFP-FANCI, mCherry-UHRF1 cells. Cells were treated with TMP and microirradiated at the indicated areas (white arrows) and followed over time. Quantification showing relative intensities of the irradiated areas. Mean ± SEM. A total of 5 cells were quantified for each chart. Scale bar, 10 µm. F) Combination of quantification data from (E), showing recruitment of EGFP-FANCI in control cells and in cells where WRNIP1 is depleted. ** indicates that $p < 0.01$, *** indicates that $p < 0.001$. Mean ± SEM.

Figure S4

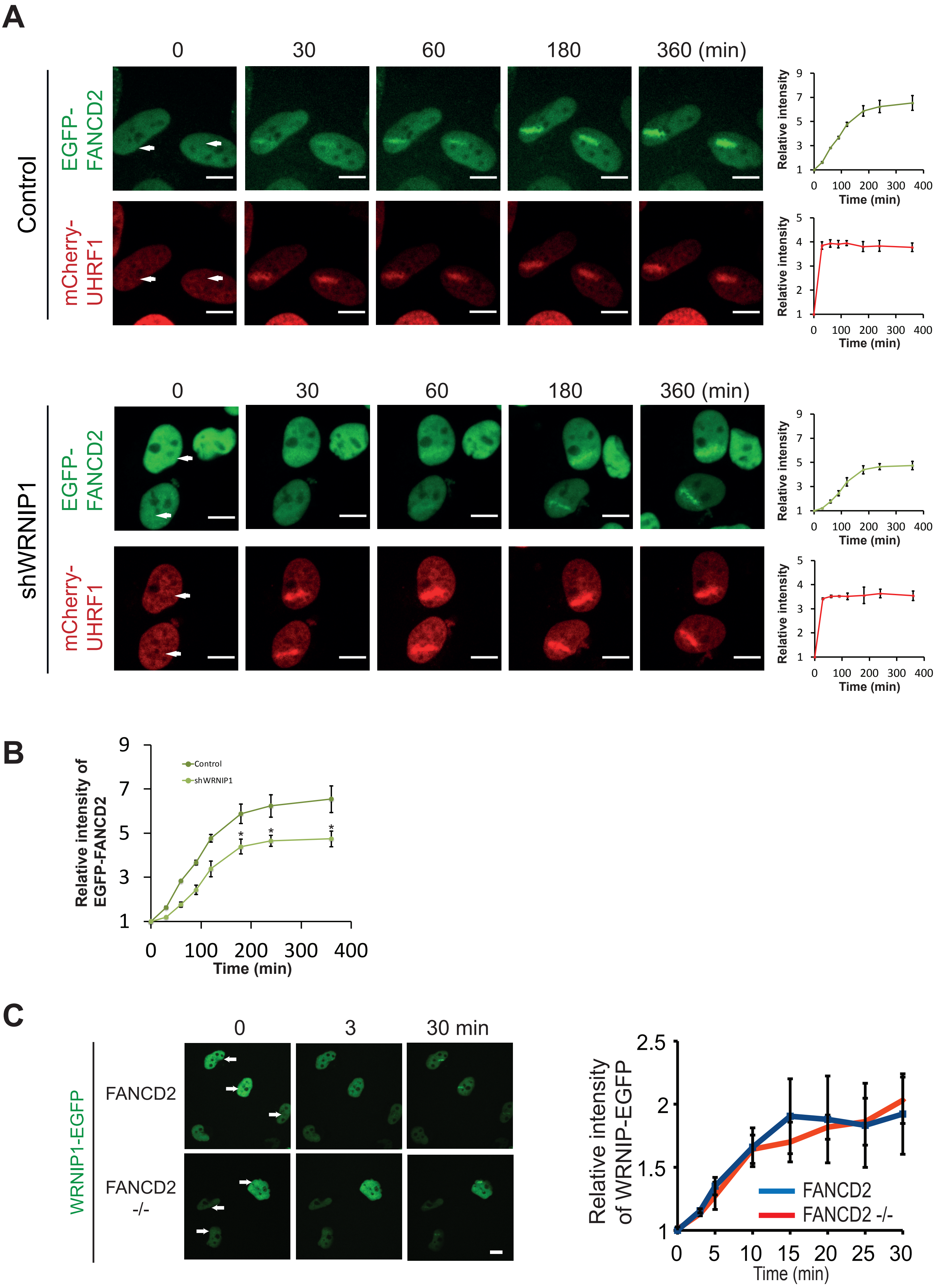

(legend on next page)

Figure S4. WRNIP1 is recruited to ICLs. Relating to Figure 3. A) Live-cell imaging of HeLa-EGFP-FANCD2, mCherry-UHRF1 cells. Cells were treated with TMP and microirradiated at the indicated areas (white arrows) and followed over time. Quantification showing relative intensities of the irradiated areas. A total of 4 cells were quantified for each chart. Mean ± SEM. Scale bar, 10 µm. B) Combination of quantification data from (A), showing recruitment of EGFP-FANCD2 in control cells and in cells where WRNIP1 is depleted. Mean ± SEM. * indicates that $p < 0.05$. C) Live-cell imaging of Flp-in T-Rex HeLa-WRNIP1-EGFP and Flp-in T-Rex HeLa FANCD2 -/- +WRNIP1-EGFP cells. Cells were treated with Doxycycline and TMP and microirradiated at the indicated areas (white arrows) and followed over time. Scale bar, 10 µm. Quantification showing relative intensities of the irradiated areas. Data represent 2 independent experiments (n=2) and 14 cells in total were quantified, 9 control cells and 5 FANCD2 deficient cells. Mean ± SEM.

Figure S5

A

Sites	Frequency
K62	2
K70	5
K141	2
K287	2
K301	2
K310	2
K464	2
K482	1
K627	1
K661	1

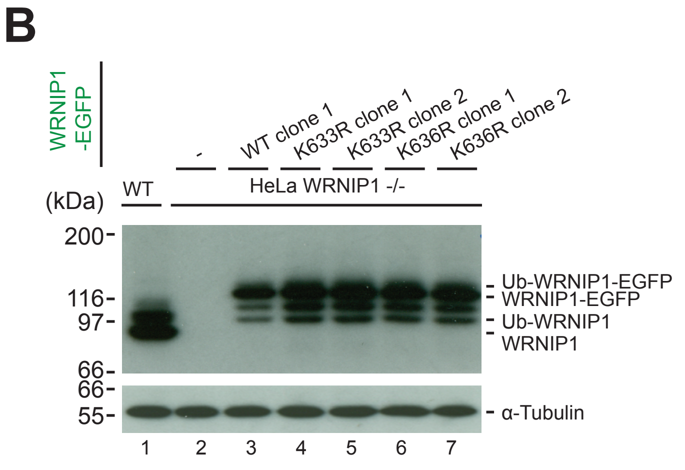

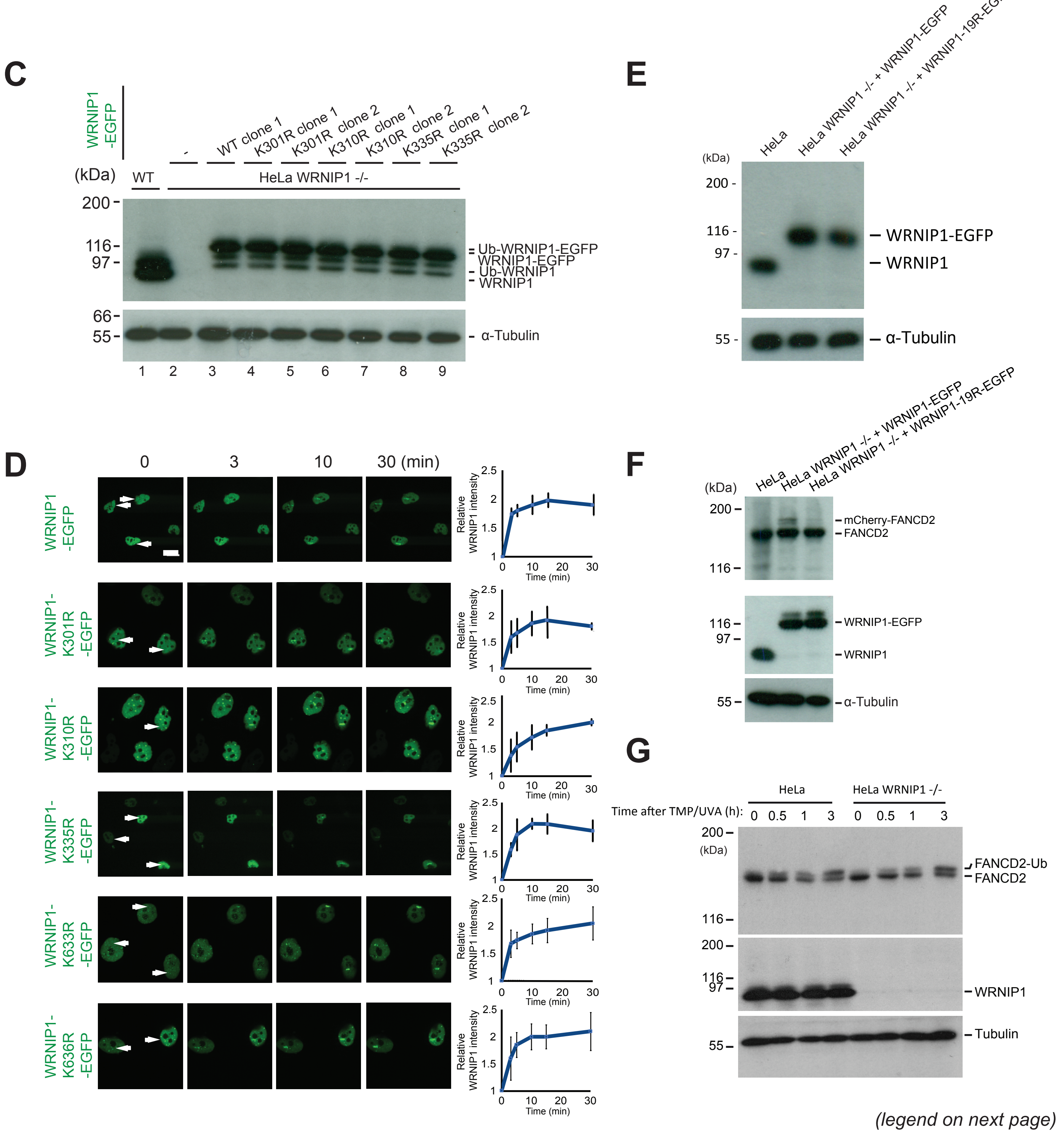

(legend on next page)

Figure S5. WRNIP1 is ubiquitinated. Relating to Figure 4. A) Details of Gly-Gly-containing peptides identified from WRNIP1. B-C) The CRISPR/Cas9 technique was used to disrupt the *WRNIP1* gene in HeLa cells. The indicated derivatives of WRNIP1-EGFP were then expressed in the WRNIP1 deficient cell line. Immunoblot analysis. D) Live-cell imaging of Flp-in T-Rex HeLa WRNIP1 -/- cells complemented with the indicated derivatives of WRNIP1-EGFP. Cells were treated with Doxycycline and TMP and microirradiated at the indicated areas (white arrows) and followed over time. Quantification showing relative intensities of the irradiated areas. Data represent 2 independent experiments (n=2) and the following number of cells were analysed: 7 cells for K301, 6 cells for K310R, 6 cells for K335R, 6 cells for K633R and 5 cells for K636R. Mean ± SEM. Scale bar, 10 µm. E) Immunoblot analysis showing expression of WRNIP1-EGFP and WRNIP1-19R-EGFP in HeLa WRNIP1 -/- cells. F) Immunoblot analysis showing expression of WRNIP1-EGFP or WRNIP1-19R-EGFP, and mCherry-FANCD2 stably expressed in HeLa WRNIP1 -/- cells. G) Immunoblot analysis of FANCD2 and WRNIP1 in HeLa and HeLa WRNIP1 -/- cells following treatment with TMP/UVA.

Figure S6

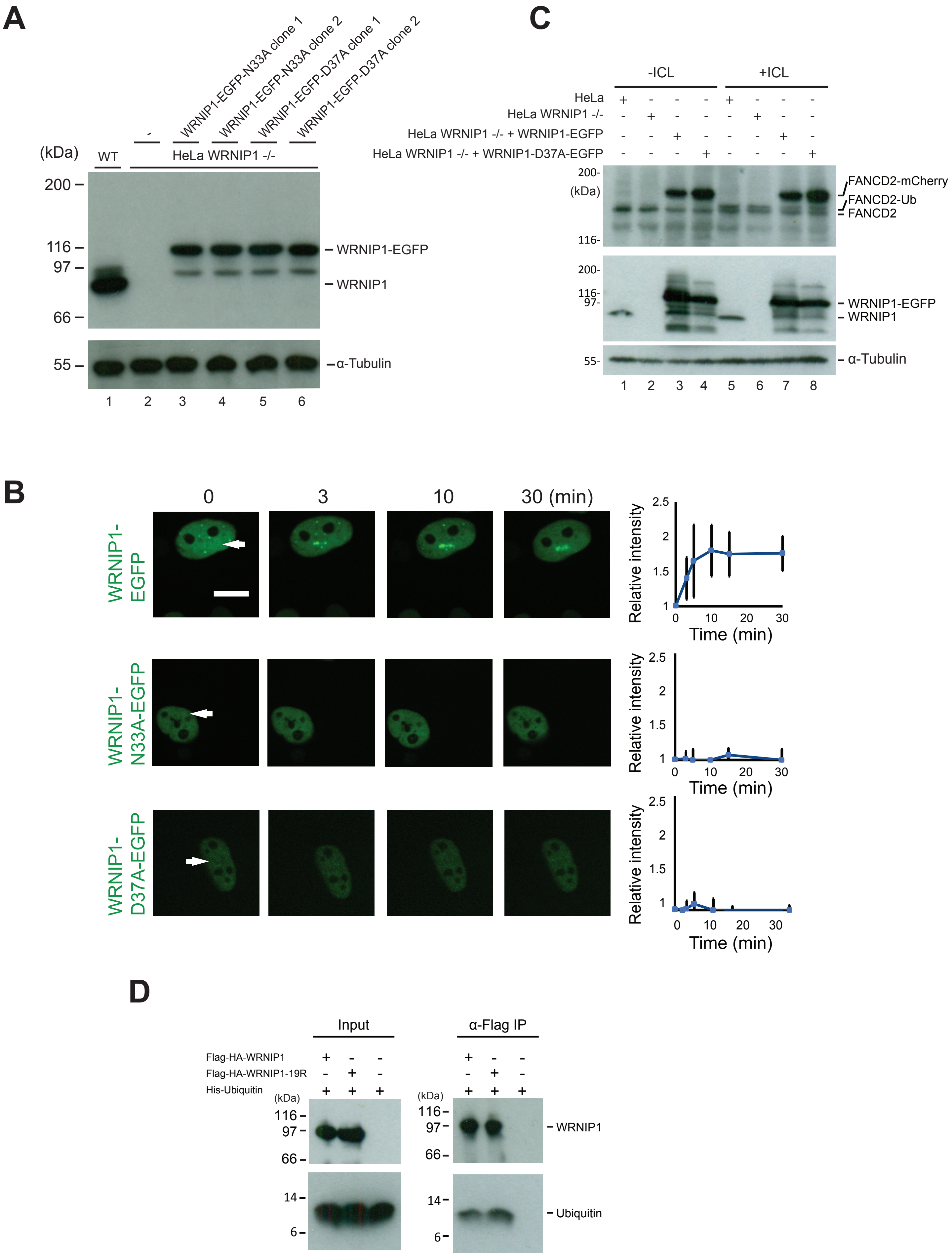

(legend on next page)

Figure S6. The UBZ domain of WRNIP1 is necessary for its ICL repair activity. Relating to Figure 6. A) The CRISPR/Cas9 technique was used to disrupt the WRNIP1 gene in HeLa Flp-in T-Rex cells. The resulting Flp-in T-Rex HeLa-WRNIP1 -/- cells were then complemented with the indicated derivatives of WRNIP1-EGFP. Cells were treated with Doxycycline. Immunoblot analysis using the indicated antibodies. B) Live-cell imaging of Flp-in T-Rex HeLa-WRNIP1-EGFP, HeLa-WRNIP1-N22A-EGFP and HeLa-WRNIP1-D37A-EGFP cells. Cells were treated with Doxycycline and TMP and microirradiated at the indicated areas (white arrows) and followed over time. Quantification showing relative intensities of the irradiated areas. Data represent 2 independent experiments (n=2) and 5 cells in total were quantified for WRNIP1-EGFP, 9 cells in total were quantified for WRNIP1-N33A-EGFP and 5 cells in total were quantified for WRNIP1-D37A-EGFP. Mean ± SEM. Scale bar, 10 μm. C) Immunoblot analysis showing stable expression of mCherry-FANCD2, WRNIP1-EGFP or WRNIP1-D37A-EGFP in HeLa WRNIP1 -/- cells in untreated cells, or in cells treated with TMP/UVA 3h prior to harvest to introduce ICLs. D) In vitro protein-protein interaction assay showing a direct interaction between WRNIP1 and Ubiquitin, and WRNIP1-19R and Ubiquitin. Immunoblot analysis using anti-WRNIP1 and anti-Ubiquitin antibodies as indicated.

Figure S7

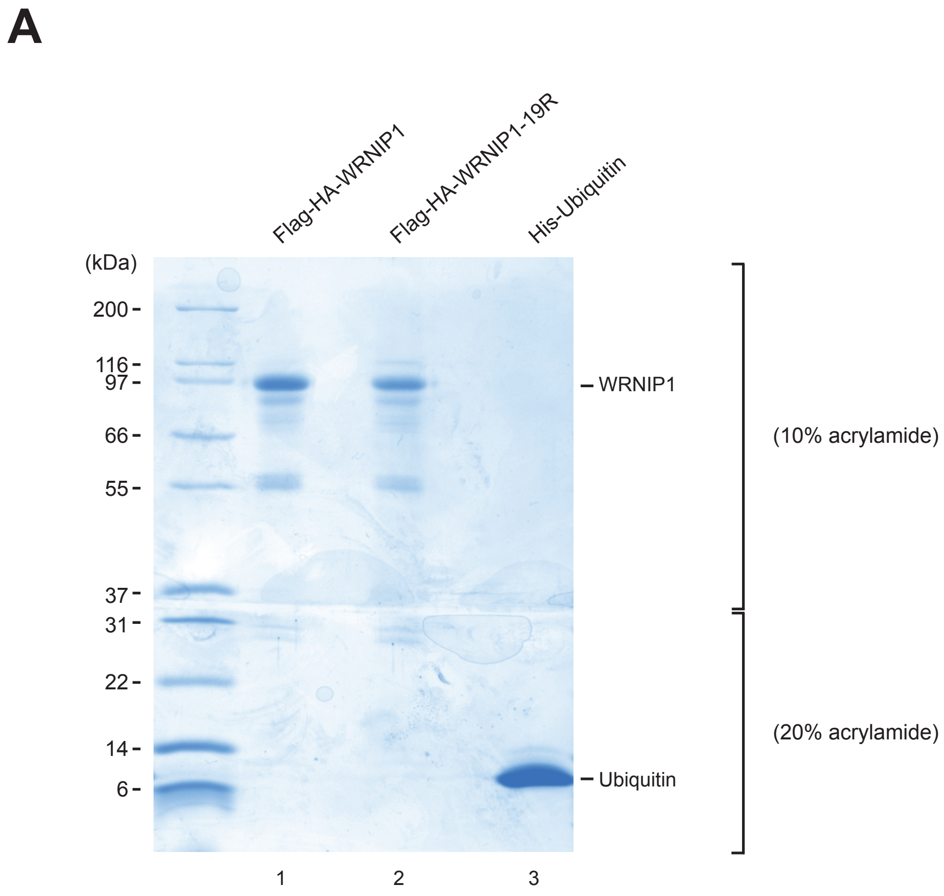

Figure S7. The UBZ domain of WRNIP1 is necessary for its ICL repair activity. Relating to Figure 6. A) Coomassie blue staining of the recombinant proteins used in the *in vitro* protein-protein interaction assay shown in Figure 6E. The upper part of the separation gel contained 10% acrylamide and lower part contained 20% acrylamide.
