# Supplementary material for: WRNIP1 Is Recruited to DNA Interstrand Crosslinks and Promotes Repair
Source: Cell Rep. 2020 Jul 7;32(1):107850. doi: 10.1016/j.celrep.2020.107850 (PMC7351111; doi:10.1016/j.celrep.2020.107850)
Supplement: Document S2. Article plus Supplemental Information [file mmc4.pdf]

Cell Reports

Article

WRNIP1 Is Recruited to DNA Interstrand Crosslinks and Promotes Repair

Graphical Abstract

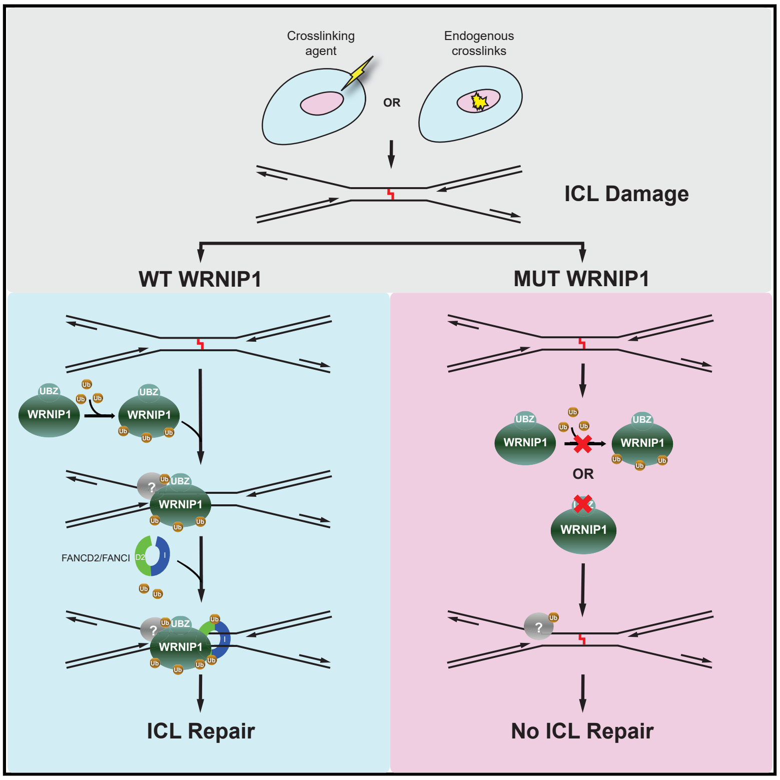

Authors

Anna Socha, Di Yang, Alicja Bulsiewicz, ..., Ronghu Wu, Steven P. Gygi, Martin A. Cohn

Correspondence

martin.cohn@bioch.ox.ac.uk

In Brief

Socha et al. report the identification of several proteins present in a FANCD2-containing complex. WRNIP1 is one subunit that is recruited to ICLs and facilitates chromatin loading of the FANCD2/FANCI complex, integral to the Fanconi anemia pathway.

Highlights

- Multiple proteins are identified in a FANCD2 protein complex, including WRNIP1
- WRNIP1 is recruited to DNA interstrand crosslinks and promotes DNA repair
- Recruitment of WRNIP1 facilitates loading of the FANCD2/FANCI complex onto DNA
- Ubiquitination of WRNIP1 and its UBZ domain are required for DNA repair

Socha et al., 2020, Cell Reports *32*, 107850
July 7, 2020 © 2020 The Author(s).
https://doi.org/10.1016/j.celrep.2020.107850

CellPress

Cell Reports

CellPress
OPEN ACCESS

Article

WRNIP1 Is Recruited to DNA Interstrand Crosslinks and Promotes Repair

Anna Socha,[1,3] Di Yang,[1,3] Alicja Bulsiewicz,[1,3] Kelvin Yaprianto,[1] Marian Kupculak,[1] Chih-Chao Liang,[1] Andreas Hadjicharalambous,[1] Ronghu Wu,[2] Steven P. Gygi,[2] and Martin A. Cohn[1,4,*]

[1]Department of Biochemistry, University of Oxford, Oxford OX1 3QU, UK
[2]Department of Cell Biology, Harvard Medical School, Boston, MA 01125, USA
[3]These authors contributed equally
[4]Lead Contact
*Correspondence: martin.cohn@bioch.ox.ac.uk
https://doi.org/10.1016/j.celrep.2020.107850

SUMMARY

The Fanconi anemia (FA) pathway repairs DNA interstrand crosslinks (ICLs). Many FA proteins are recruited to ICLs in a timely fashion so that coordinated repair can occur. However, the mechanism of this process is poorly understood. Here, we report the purification of a FANCD2-containing protein complex with multiple subunits, including WRNIP1. Using live-cell imaging, we show that WRNIP1 is recruited to ICLs quickly after their appearance, promoting repair. The observed recruitment facilitates subsequent recruitment of the FANCD2/FANCI complex. Depletion of WRNIP1 sensitizes cells to ICL-forming drugs. We find that ubiquitination of WRNIP1 and the activity of its UBZ domain are required to facilitate recruitment of FANCD2/FANCI and promote repair. Altogether, we describe a mechanism by which WRNIP1 is recruited rapidly to ICLs, resulting in chromatin loading of the FANCD2/FANCI complex in an unusual process entailing ubiquitination of WRNIP1 and the activity of its integral UBZ domain.

INTRODUCTION

Crosslinks holding the Watson-Crick strands of DNA together are highly toxic to cells. One of the most studied pathways repairing this type of DNA damage in humans is the Fanconi anemia (FA) DNA repair pathway. Individuals with an inactive FA pathway suffer from the FA disease, which is a rare, autosomal, recessive disorder with incidence estimated to be between 1/200,000 and 1/400,000 in the general population (Dong et al., 2015). Although FA primarily presents as a bone marrow failure disease, it has been associated with several other clinical manifestations, such as immune deficiency (Fagerlie and Bagby, 2006), endocrine dysfunction, osteoporosis, and cancer (Giri et al., 2007).

The molecular basis for the phenotype in patients is mutation of any of the 22 FA genes associated with DNA crosslink repair, although it remains a subject of discussion as to whether all clinical manifestations of FA stem directly from impaired DNA damage repair or from other not fully investigated causes (Niraj et al., 2019). Not all FA genes have been identified, and the mechanism of repair is not fully understood (Lopez-Martinez et al., 2016).

Controlled ubiquitination and deubiquitination of several interstrand crosslink (ICL) repair proteins is essential for the pathway. For instance, both the FANCD2 and the FANCI proteins are monoubiquitinated, an event essential to their repair functions. In addition, the presence of ubiquitin binding domains, such as the UBZ domain, plays an important function for ICL repair. For instance, SLX4 harbors such a domain, which is important for its repair function. The WRNIP1 protein both harbors a UBZ domain and is ubiquitinated (Bish et al., 2008), but it has not yet been linked to ICL repair. The protein is 665 aa long and possesses an AAA+ ATPase domain. Its UBZ domain is capable of binding both monoubiquitin (Crosetto et al., 2008) and polyubiquitin (Bish and Myers, 2007). Apart from being modified by ubiquitination, WRNIP1 is sumoylated (Bish and Myers, 2007), but the exact functions of these post-translational modifications remain elusive.

The ATPase domain consists of Walker A and Walker B motifs that trap and hydrolyze ATP. This domain was shown to interact with WRN helicase (Kawabe et al., 2006). Finally, the C terminus of the protein contains a predicted leucine zipper domain, which consists of two putative leucine zipper motifs between 519 and 655 aa.

WRNIP1 was first identified by a yeast two-hybrid assay as a binding partner of the mouse WRN helicase (Kawabe et al., 2001). In the same study, it was confirmed that this interaction is direct, which raised the question of whether WRNIP1 and WRN act in the same pathway. This hypothesis was later disproved (Kawabe et al., 2006). WRNIP1 has also been reported to interact with three of the four subunits of DNA polymerase delta, presumably stimulating its activity (Tsurimoto et al., 2005), as well as with polymerase eta (Yoshimura et al., 2014). A physical interaction with the E3 ligase RAD18 has been shown (Yoshimura et al., 2009), and WRNIP1 was found to interact with the ataxia telangiectasia mutated (ATM) cofactor ATMIN (Kanu et al., 2016).

This is an open access article under the CC BY license (http://creativecommons.org/licenses/by/4.0/).

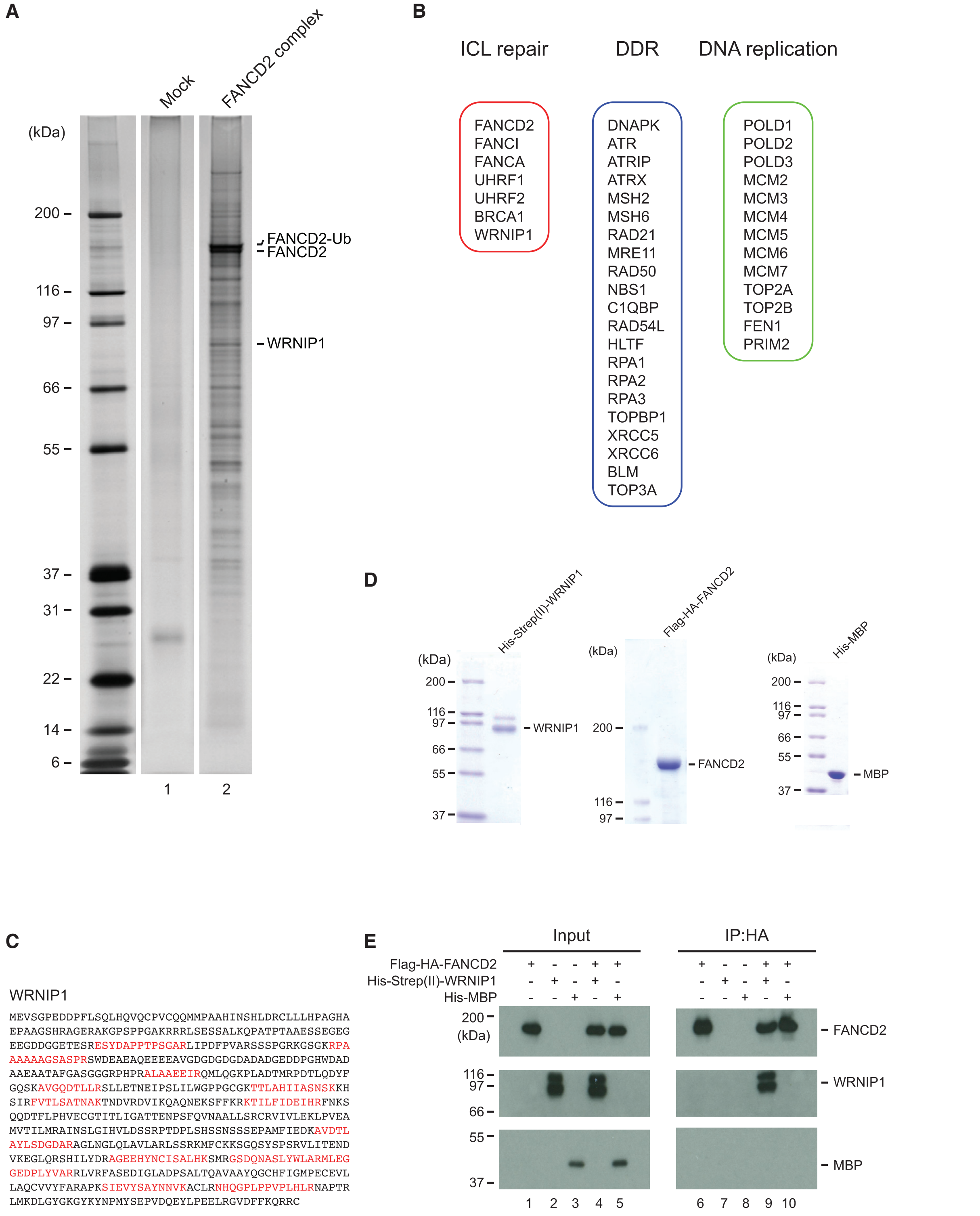

Figure 1. Purification of the FANCD2 Protein Complex Containing WRNIP1

(A) The FANCD2 complex was purified from HeLa cells. Proteins were resolved via SDS–PAGE and visualized using silver stain.

(B) Three groups of proteins identified in the purified complexes. DDR refers to DDR proteins not primarily associated with ICL repair. See Table S1 for full list of proteins.

(legend continued on next page)

The best characterized homolog of WRNIP1 in other eukaryotes is the yeast protein Mgs1 (maintenance of genome stability), which interacts physically and functionally with Pol31, a subunit of polymerase delta (Vijeh Motlagh et al., 2006) and was reported to bind PCNA (Saugar et al., 2012). Although genetic analyses of Mgs1 have shown that the protein is required for genome stability (Hishida et al., 2001), the role of WRNIP1 remains elusive. WRNIP1 shares sequence similarity with the bacterial replication protein RarA (Lau et al., 2003; Sherratt et al., 2004). WRNIP1 also shares sequence similarity with the bacterial RuvB protein, which also exhibits ATPase activity, forms a hexameric ring complex, and is known to drive DNA strand migration at Holliday junction structures in complex with the RuvA protein (Yamada et al., 2002). It was reported that WRNIP1 can form a homo-octameric complex under certain conditions (Tsurimoto et al., 2005). These data were obtained using WRNIP1 expressed and purified from insect cells. So far, no functional involvement of WRNIP1 in Holliday junction migration in eukaryotes has been reported.

It has been suggested that WRNIP1 accumulates at DNA damage sites in response to double-stranded breaks (Nomura et al., 2012) and that UVC treatment causes an increase in the number of WRNIP1 nuclear foci (Crosetto et al., 2008). Microscopy data showed that WRNIP1 foci tend to overlap with replication factories, which reinforces the hypothesis of a function at replication forks (Crosetto et al., 2008). *In vitro* analysis has shown that WRNIP1 binds to forked DNA that mimics stalled replication forks in an ATP-dependent manner (Yoshimura et al., 2009), as well as DNA with single-stranded DNA (ssDNA) overhang (Kanamori et al., 2011). It has been reported that WRNIP1 plays a role in protecting stalled replication forks from degradation and promoting fork restart (Leuzzi et al., 2016; Marabitti et al., 2020; Porebski et al., 2019). The first of these studies described a process entailing stabilization of RAD51 on ssDNA by WRNIP1, thereby preventing uncontrolled MRE11-mediated degradation of stalled replication forks. The study suggests that although the protection does not require the ATPase activity of WRNIP1, this activity is needed for the recovery of the stalled fork. The second study reported stabilization of the stalled replication fork by protection against MUS81- and EME1-mediated degradation. In addition, WRNIP1 was recently found enriched at chromosomal fragile sites, suggesting a role in maintaining their stability (Pladevall-Morera et al., 2019).

Here we report the identification of a new role of WRNIP1, functioning in the FA pathway to repair DNA ICLs. Using live-cell imaging, we demonstrate that WRNIP1 is specifically recruited to ICLs quickly after their appearance in the genome. Importantly, the UBZ domain of WRNIP1, as well as its own ubiquitination, is critical for this process. WRNIP1 physically interacts with the FANCD2/FANCI complex and promotes its recruitment to ICLs.

RESULTS

Purification of a FANCD2 Complex Containing WRNIP1 as a Subunit

To identify putative novel ICL repair proteins, we purified FANCD2, together with associated proteins, as protein complexes from HeLa cells. Functional fusion protein of FANCD2 tagged by Flag and hemagglutinin (HA) (Flag-HA-FANCD2) (Liang et al., 2016) was stably expressed in HeLa cells. Cells were treated with mitomycin C (MMC) to introduce ICLs into the genome, triggering an activation of the FA pathway and ICL repair. Nuclear extract was prepared and Flag-HA-FANCD2 was purified, together with associated proteins, by a modified version of a previously reported two-step immunoaffinity purification scheme (Cohn et al., 2007). SDS-PAGE analysis of the purified complexes revealed the presence of multiple polypeptides (Figure 1A, lane 2). No polypeptides were observed in a mock purification from HeLa cells not expressing Flag-HA-FANCD2 (Figure 1A, lane 1), indicating that the polypeptides copurified with Flag-HA-FANCD2 were bona fide subunits of FANCD2 complexes. To identify the subunits of the purified FANCD2 complex, we repeated the purification on a larger scale, now 6 L of suspension HeLa culture, and concentrated the purified protein complexes by trichloroacetic acid (TCA) precipitation. Precipitated proteins were identified by tandem mass spectrometry (MS/MS) analysis. As expected, several DNA repair proteins that have been shown to either physically or functionally interact with FANCD2 and the FA pathway and ICL repair were identified. Examples of these are FANCI, FANCA, UHRF1, and BRCA1 (Figure 1B; see Table S1 for a complete list of proteins). Homologous recombination (HR) is an integral part of ICL repair via the FA pathway. Several HR factors, such as MRE11, RAD50, and BLM, were identified as subunits. We also identified several DNA replication factors, such as MCM2-7 and TOP2A, in accordance with previous reports (Lossaint et al., 2013). In addition to these expected subunits, several proteins that have not been connected to ICL repair were found. One such protein, WRNIP1, was identified by 21 peptides (Figure 1C) and can be observed on silver stain of the protein complex (Figure 1A).

We hypothesized that WRNIP1 might interact directly with FANCD2. To test this, we purified both proteins to homogeneity (Figure 1D) and assessed their abilities to interact with each other *in vitro*. WRNIP1 formed a direct protein-protein interaction with FANCD2, whereas no interaction was observed between MBP and FANCD2, underscoring the specificity of the interaction (Figure 1E). Because the FANCD2/FANCI complex undergoes a conformational change upon monoubiquitination, it is plausible that the interaction with WRNIP1 could be altered as a result. Therefore, we assessed the interaction of WRNIP1 with the FANCD2/FANCI complex and the ubiquitin-FANCD2/FANCI (Ub-FANCD2/FANCI) complex. We found that WRNIP1 interacted with both complexes (Figure S1A). However, it cannot be excluded that the interaction of WRNIP1 and the FANCD2/FANCI complex is regulated upon DNA damage in live cells, in

(C) Positions of peptides (shown in red) originating from WRNIP1 following MS/MS analysis of purification in (A).
(D) Coomassie blue staining of the recombinant proteins used in the *in vitro* protein-protein interaction assay shown in (E).
(E) Immunoblot analysis of the *in vitro* protein-protein interaction assay showing direct interaction between WRNIP1 and FANCD2.

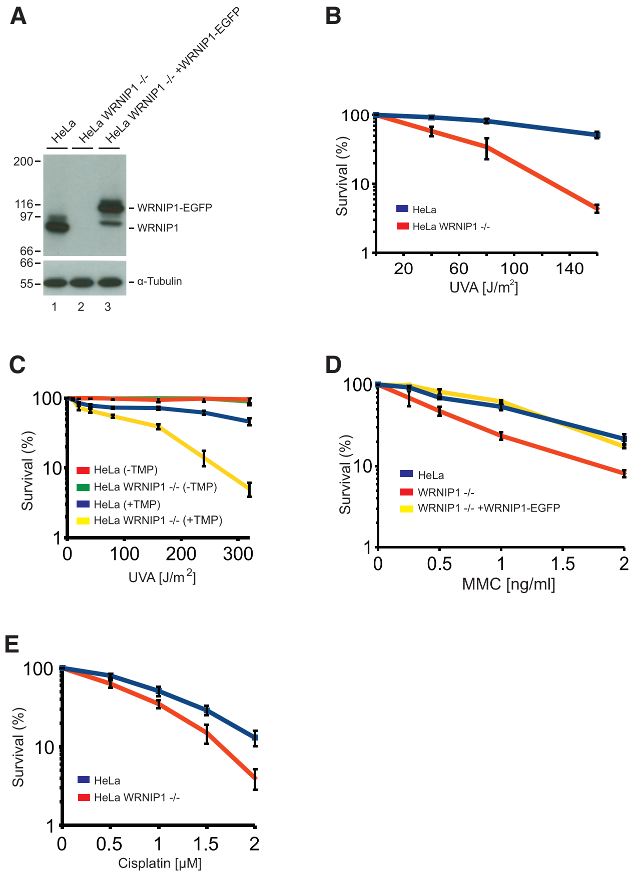

Figure 2. WRNIP1 Is Important for ICL Repair

(A) CRISPR-Cas9 technique was used to disrupt the *WRNIP1* gene in HeLa cells, creating a WRNIP1-deficient HeLa cell line. HeLa WRNIP1$^{-/-}$ cells were complemented by stable expression of WRNIP1-EGFP.

(B) Clonogenic survival assay of HeLa and HeLa WRNIP1$^{-/-}$ cells in response to TMP/UVA (mean ± SEM, n = 3).

(C) Clonogenic survival assay of HeLa and HeLa WRNIP1$^{-/-}$ cells in response to TMP/UVA or only UVA as a negative control (mean ± SEM).

(D) Clonogenic survival assay of HeLa, HeLa WRNIP1$^{-/-}$, and HeLa WRNIP1$^{-/-}$ complemented with WRNIP1-EGFP cells in response to MMC (mean ± SEM, n = 3).

(E) Clonogenic survival assay of HeLa and HeLa WRNIP1$^{-/-}$ cells in response to cisplatin (mean ± SEM).

particular when the proteins are docked on DNA. Further experimentation might shed light on this.

WRNIP1$^{-/-}$ Cells Are Sensitive to ICL-Inducing Agents

Given the presence of WRNIP1 in the FANCD2 complex and that the two proteins interact directly, we decided to determine whether WRNIP1 is functionally important for the cellular response to ICLs. To test this directly, we disrupted the *WRNIP1* gene in HeLa Flp-in T-Rex cells using CRISPR-Cas9 genome editing. The resulting cell line was deficient for WRNIP1 (Figure 2A). We then subjected these cells to a clonogenic survival assay, assessing their ability to survive after the introduction of increasing amounts of ICLs formed by trioxsalen (TMP)/UVA treatment. We found that depletion of WRNIP1 sensitized cells to ICLs when compared with control cells (Figure 2B). In addition, we generated a second independent cell line with the *WRNIP1* gene disrupted and observed identical results (Figure S1B). To ensure that the observed phenotype results from ICLs formed by TMP/UVA and is not an effect of UVA alone, we assessed the sensitivity of the cells after treatment with increasing doses of UVA in the absence of TMP. No significant reduction in viability was observed, confirming our conclusion that WRNIP1 contributes to the repair of ICLs formed by TMP/UVA (Figure 2C).

Given the observed sensitivity of WRNIP1-deficient cells to ICLs introduced by TMP/UVA, we wished to test whether WRNIP1 is generally important for the cellular response to ICLs. We therefore subjected HeLa WRNIP1$^{-/-}$ cells, a third independent clone, to a clonogenic survival assay in which we introduced ICLs caused by MMC. These experiments demonstrated that WRNIP1 is also required for the cellular response to this type of ICL (Figure 2D). These data were confirmed using an independently obtained clone, showing similar results (Figure S1C). Importantly, expressing WRNIP1-EGFP in the deficient cells (Figure 2A) restored the resistance to ICLs to the level observed in control cells, ruling out off-target and clonal effects (Figure 2D).

We also observed sensitivity of WRNIP1-deficient cells to cisplatin, which forms yet another type of ICL (Figure 2E). UVC and hydroxyurea treatments, which do not introduce ICLs, did not affect survival of the WRNIP1-deficient cell line (Figures S1D and S1E).

Altogether, our data show that WRNIP1 is necessary for the cellular response to a range of ICLs.

WRNIP1 Cooperates with FANCD2 in ICL Repair

Our data show that the WRNIP1 protein is functionally important for ICL repair. The FANCD2 protein is central to the FA pathway repairing ICLs in human cells. Therefore, we sought to determine whether depleting WRNIP1 further sensitizes HeLa cells deficient in FANCD2. We created HeLa cell lines depleted of FANCD2, WRNIP1, or both using CRISPR-Cas9 (Figure 3A). The resulting cell lines were subjected to a clonogenic survival assay in which we introduced an increasing number of ICLs using TMP/UVA. As expected, depletion of FANCD2 sensitized cells significantly (Figure 3B). When we depleted WRNIP1 in these FANCD2-deficient cells, we observed no further sensitization. Similar results were seen when ICLs were introduced using MMC (Figure 3C). To rule out clonal effects, we repeated the experiment using a second independent cell clone (Figure S2A) and obtained identical results (Figure S2B). These data suggest that the two proteins interact functionally and place the WRNIP1 protein in the FA ICL repair pathway.

WRNIP1 Is Recruited to ICLs in Live Cells

The functional importance of WRNIP1 in ICL repair led us to speculate that the protein might be recruited to ICLs in chromosomes. To test this directly, we applied live-cell microscopy. We expressed EGFP-tagged WRNIP1 in HeLa Flp-in T-Rex cells, ensuring that the fusion protein is expressed at the same level as endogenous WRNIP1 (Figure S3A). The cells were then treated with TMP and ICLs were introduced in a local area of the nucleus using a UVA laser while the cells were observed

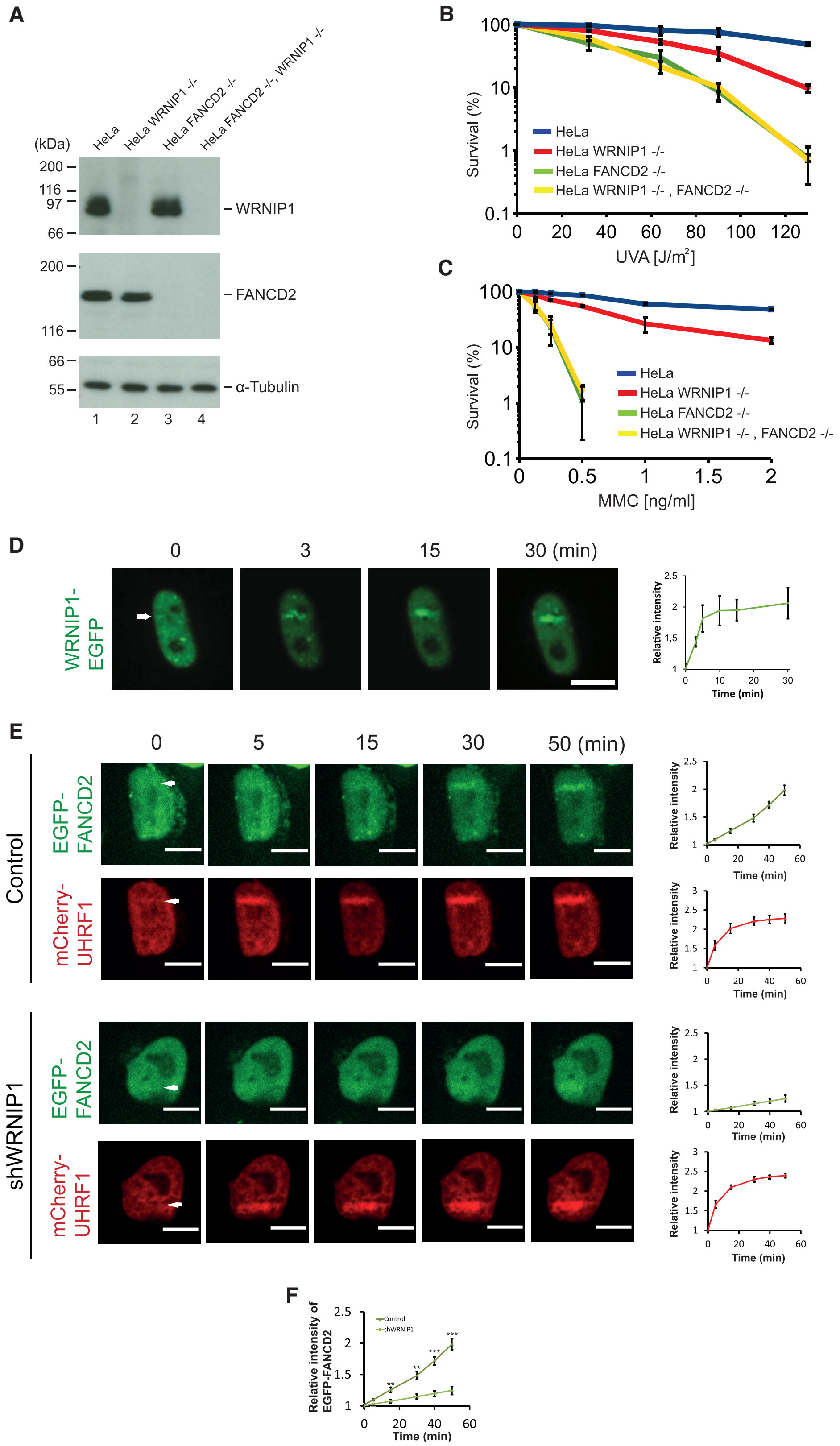

(legend on next page)

using a microscope. We observed strong recruitment of WRNIP1-EGFP to ICLs within minutes of their introduction into the chromosomes (Figure 3D).

To gain insight into how WRNIP1 functions in ICL repair, we decided to determine the kinetics of WRNIP1 recruitment to ICLs relative to the well-characterized kinetics of FANCD2 recruitment and to test whether recruitment of one protein affects recruitment of the other. Whereas WRNIP1 was recruited relatively quickly (Figure 3D), the kinetics of the FANCD2 recruitment appeared to be slower (Figure 3E). The difference in timing of recruitment suggested that WRNIP1 might be recruited to ICLs before FANCD2. If WRNIP1 is recruited to ICLs before FANCD2, recruitment of WRNIP1 may affect the subsequent recruitment of FANCD2. Such a functional relationship would be in good agreement with the data showing that depletion of WRNIP1 in cells from which FANCD2 is depleted does not further sensitize cells to ICLs (Figures 3B and 3C). To test this hypothesis directly, we depleted WRNIP1 in cells stably expressing EGFP-FANCD2 and mCherry-UHRF1, as a marker of ICLs (Figure S3B), and assessed the recruitment of FANCD2 to ICLs compared with control cells. Depletion of WRNIP1 caused a clear reduction in FANCD2 recruitment, whereas the recruitment of UHRF1 as a control for the generation of ICLs was unaffected (Figures 3E and 3F). To test whether WRNIP1 also assists FANCD2 recruitment in response to other types of ICLs, we determined its effect on FANCD2 foci formation in response to MMC using live-cell imaging. We observed that depletion of WRNIP1 suppresses the accumulation of FANCD2 nuclear foci over time (Figures S3C and S3D).

FANCD2 functions as a heterodimer with the FANCI protein in ICL repair. Therefore, we tested whether WRNIP1 is required for the recruitment of EGFP-FANCI (Figure S3B) to ICLs. As observed for FANCD2, the recruitment of FANCI is reduced when WRNIP1 is depleted (Figures S3E and S3F).

Because the recruitment of both FANCD2 and FANCI was reduced upon depletion of WRNIP1 in relatively short timescales using live-cell imaging, we decided to test whether cells would be able to support full FANCD2 recruitment over longer periods in the absence of WRNIP1. Therefore, we monitored EGFP-FANCD2 recruitment over 6 h. Even during such long periods, we observed a reduction in FANCD2 recruitment (Figures S4A and S4B). These data are in good agreement with other longer-timescale experiments, clonogenic survival assays, demonstrating that depletion of WRNIP1 sensitized cells to ICLs (Figures 3B and 3C).

To test whether FANCD2 affects the recruitment of WRNIP1, we depleted FANCD2 in cells expressing WRNIP1-EGFP (Figure S3A) and assessed whether the recruitment of WRNIP1 was affected. We observed no change in the recruitment of WRNIP1 in the absence of FANCD2 (Figure S4C).

Altogether, these data show that WRNIP1 is recruited to ICLs and that this recruitment facilitates the recruitment of the FANCD2/FANCI complex, potentially via a direct protein-protein interaction between the two proteins.

Ubiquitination of WRNIP1 Is Required for Its Recruitment to ICLs

We noticed that WRNIP1 migrates as two species in SDS-PAGE: a major fast-migrating form and a less abundant slower-migrating form (for instance, Figures 1D and 2A). We hypothesized that the slow-migrating form of WRNIP1 could be a result of ubiquitination, supported by an earlier report on WRNIP1 (Bish et al., 2008). We examined both species by immunoblot analysis using specific antibodies against WRNIP1, 6xHis, and ubiquitin. Although both species were detected using the first two antibodies, only the upper species was detected by the anti-ubiquitin antibody, indicating that WRNIP1 indeed is ubiquitinated (Figure 4A). The anti-ubiquitin antibody also detected less abundant species with a higher molecular weight. Because these species could not be detected using the antibodies against WRNIP1 or 6xHis, they are likely low in abundance compared with the faster-migrating species of WRNIP1.

Ubiquitination often plays key regulatory roles in DNA repair. Therefore, we decided to investigate the nature of WRNIP1 ubiquitination. To determine which lysine or lysines in WRNIP1 were modified by ubiquitin, we stably expressed and purified Flag-HA-WRNIP1 from HeLa S3 cells. Flag-HA-WRNIP1 was purified as the two described characteristic species, along with several interacting proteins (Figure 4B).

We excised the modified form of WRNIP1 from the gel (Figure 4C) and identified lysines modified by ubiquitin using mass spectrometry, detecting the characteristic residual Gly-Gly signature peptide following trypsinization. Surprisingly, not one but several ubiquitinated lysines were identified in this analysis (Figures 4D and S5A; Table S2). Because the difference in molecular weight between the upper and the lower forms of WRNIP1 is not large enough for all identified lysines to be mono-ubiquitinated simultaneously, the data indicate that the upper form of WRNIP1 is heterogenous. Most likely, this upper form

Figure 3. WRNIP1 Is Recruited to ICLs

(A) CRISPR-Cas9 technique was used to disrupt the *WRNIP1* and *FANCD2* genes in HeLa cells, creating HeLa WRNIP1$^{-/-}$ (clone 1), HeLa FANCD2$^{-/-}$, and HeLa WRNIP1$^{-/-}$ FANCD2$^{-/-}$ (clone 1) cell lines. Immunoblot analysis.

(B) Clonogenic survival assay of the indicated cell lines in response to TMP/UVA (mean ± SEM, n = 2).

(C) Clonogenic survival assay of the indicated cell lines in response to MMC (mean ± SEM, n = 2).

(D) Live-cell imaging of Flp-in T-Rex HeLa-WRNIP1-EGFP cells. Cells were treated with doxycycline and TMP, microirradiated at the indicated areas (white arrows), and followed over time. Quantification shows relative intensities of the irradiated areas. Data represent 3 independent experiments (n = 3), and 18 cells were quantified (mean ± SEM). Scale bar, 10 μm.

(E) Live-cell imaging of HeLa cells stably expressing EGFP-FANCD2 and mCherry-UHRF1. Cells were treated with TMP, microirradiated at the indicated areas (white arrows), and followed over time. Quantification shows relative intensities of the irradiated areas. 5 cells were quantified for each chart (mean ± SEM). Scale bar, 10 μm.

(F) Combination of quantification data from (E), showing recruitment of EGFP-FANCD2 in control cells and in cells from which WRNIP1 is depleted. $^{**}p < 0.01$, $^{***}p < 0.001$ (mean ± SEM).

CellPress
OPEN ACCESS

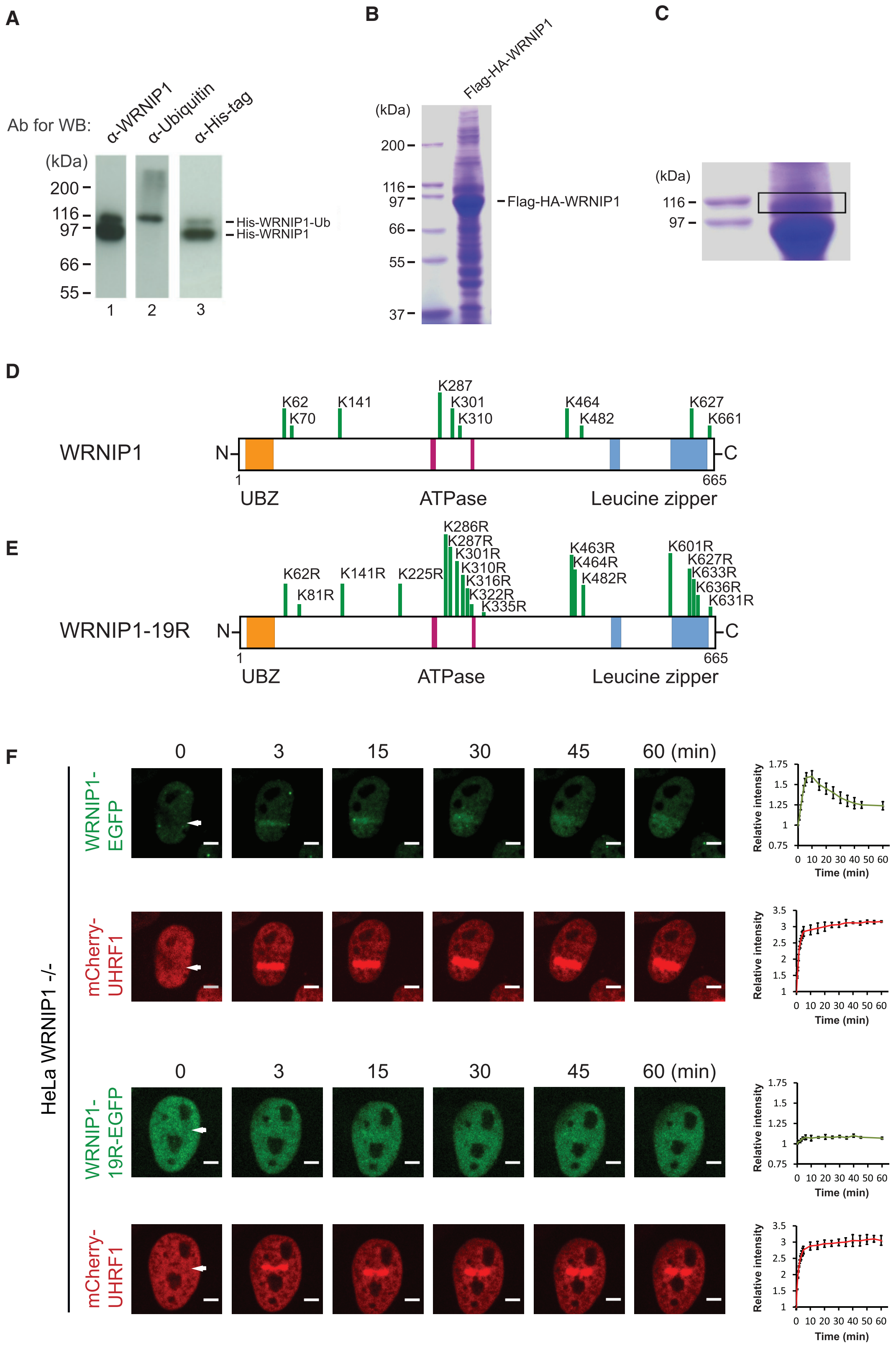

(legend on next page)

consists of WRNIP1 molecules that are mono- or multiubiquitinated at various lysine residues.

To determine whether ubiquitination of individual lysines in WRNIP1 is important for its ICL repair function, we selected the lysines that were most frequently ubiquitinated in our dataset, as well as in previous data (Bish et al., 2008). The selected lysines were mutated into arginines in WRNIP1-EGFP one at a time. The resulting derivatives of WRNIP1-EGFP were then expressed in HeLa WRNIP1$^{-/-}$ cells, ensuring protein levels comparable to endogenous WRNIP1 in HeLa cells (Figures S5B and S5C). We then tested whether the point mutations affected the ability of WRNIP1-EGFP to be recruited to ICLs using live-cell imaging. All mutant versions of WRNIP1 were recruited normally to ICLs (Figure S5D).

These data suggest that ubiquitination of individual lysines is not required for the recruitment of WRNIP1 to ICLs. It is possible that there is some redundancy in ubiquitination of residues or that ubiquitination is not strictly required for WRNIP1 recruitment. To test these hypotheses directly, we mutated into arginines the 17 most frequently ubiquitinated lysines, as determined in either the present study or a previous study (Bish et al., 2008), together with two additional lysines positioned next to two of the 17 lysines, resulting in the WRNIP1-19R derivative (Figure 4E). WRNIP1-EGFP or WRNIP1-19R-EGFP were stably expressed in HeLa WRNIP1$^{-/-}$ cells (Figure S5E). Using these cell lines, we then assessed the recruitment of WRNIP1 and WRNIP1-19R to ICLs. Although WRNIP1 was recruited rapidly and strongly to ICLs, the WRNIP1-19R protein was defective in recruitment (Figure 4F). We noticed some diffusion of the recruited WRNIP1 at later time points in some cells, causing quantification of the initially narrow irradiated area to drop mildly over time. A marker for ICLs, mCherry-UHRF1, was recruited strongly and equally in both cell lines.

Altogether, these data show that ubiquitination of WRNIP1 is required for its active recruitment to ICLs in live cells.

Ubiquitination of WRNIP1 Is Required for ICL Repair

Because recruitment of WRNIP1 promotes the recruitment of FANCD2, and ubiquitination of WRNIP1 is required for its timely recruitment to ICLs, one would expect that this ubiquitination would be necessary to promote the recruitment of FANCD2 to ICLs. To test this directly, we established HeLa cell lines from which WRNIP1 was depleted by CRISPR-Cas9, followed by stable expression of mCherry-FANCD2, and either WRNIP1-EGFP or WRNIP1-19R-EGFP (Figure S5F). The amount of residual WRNIP1 ubiquitination varied slightly depending on the experimental conditions (Figures S5E and S5F). We then assessed the ability of these proteins to be recruited to ICLs. As expected, WRNIP1-EGFP was recruited rapidly and efficiently, whereas WRNIP1-19R-EGFP was essentially defective (Figure 5A). Although cells expressing WRNIP1-EGFP displayed robust recruitment of FANCD2 to ICLs, only weak recruitment was observed in cells expressing WRNIP1-19R-EGFP (Figures 5A and 5B).

Because ubiquitination of WRNIP1 is necessary for its recruitment to ICLs, and therefore for proper recruitment of the FANCD2/FANCI complex to ICLs, we speculated that the ubiquitination reaction would also be necessary for ICL repair. To test this, we decided to determine the ability of WRNIP1-19R to functionally complement HeLa cells from which endogenous WRNIP1 is depleted. HeLa-WRNIP1$^{-/-}$ cells were stably complemented with either WRNIP1-EGFP or WRNIP1-19R-EGFP (Figure S5E). The resulting cell lines were then treated with increasing amounts of MMC, and their ability to survive was assessed using a clonogenic survival assay. Stable expression of WRNIP1-EGFP restored resistance to MMC, whereas stable expression of WRNIP1-19R-EGFP did not, suggesting that this ubiquitination of WRNIP1 is critical forICL repair (Figure 5C). We also noticed a slight increase in ubiquitination of WRNIP1 following the introduction of ICLs (Figure S5G), whereas the overall amount of ubiquitinated FANCD2 did not alter upon abrogation of WRNIP1.

Altogether, these data show that ubiquitination of WRNIP1 is required for its active recruitment to ICLs in live cells and that this facilitates local recruitment of the FANCD2/FANCI complex and promotes ICL repair.

The UBZ Domain of WRNIP1 Is Crucial for Its Ubiquitination and Recruitment to Chromatin

WRNIP1 harbors a UBZ domain at its N-terminus. UBZ domains interact with ubiquitin, either in its free form or when conjugated to other proteins. As such, these domains often mediate the regulatory functions of the proteins in which they reside. Several DNA repair proteins possess such domains, including multiple proteins in ICL repair, such as FAAP20, FAN1, and SLX4. Therefore, we speculated that the UBZ domain of WRNIP1 could be important for its ICL repair function. The crystal structure of the UBZ domain of WRNIP1 interacting with ubiquitin has been solved (Suzuki et al., 2016). Careful examination of the interphase between the two proteins revealed critical hydrogen bonds between N33 of the UBZ domain and L71 and R42 of ubiquitin, as well as an important salt bridge between D37 of the UBZ domain and R42 of ubiquitin (Figure 6A). A potential weaker interaction between A45 of the UBZ domain and K48 of ubiquitin was also observed. Alignment of the UBZ domain from WRNIP1 with the UBZ domains of other DNA repair proteins

Figure 4. Ubiquitination of WRNIP1 Is Required for Recruitment to ICLs
(A) His-tagged WRNIP1 was purified from Sf9 insect cells and analyzed by immunoblot analysis using the antibodies indicated.
(B) Coomassie blue staining of Flag-HA-WRNIP1 purified together with associated proteins from HeLa S3 cells.
(C) Magnified view of part of (B), showing the area of the gel that was excised for mass spectrometry analysis.
(D) Schematic showing locations of amino acids in WRNIP1 that were found to be modified by ubiquitin.
(E) Schematic showing locations of lysines in WRNIP1 that were mutated into arginines in WRNIP1-19R.
(F) Live-cell imaging of HeLa-WRNIP1-EGFP and HeLa-WRNIP1-19R-EGFP cells. Cells were treated with TMP, microirradiated at the indicated areas (white arrows), and followed over time. Quantification shows relative intensities of the irradiated areas. Data represent 2 independent experiments (n = 2), and 10 cells were quantified for each chart (mean ± SEM). Scale bar, 10 μm.

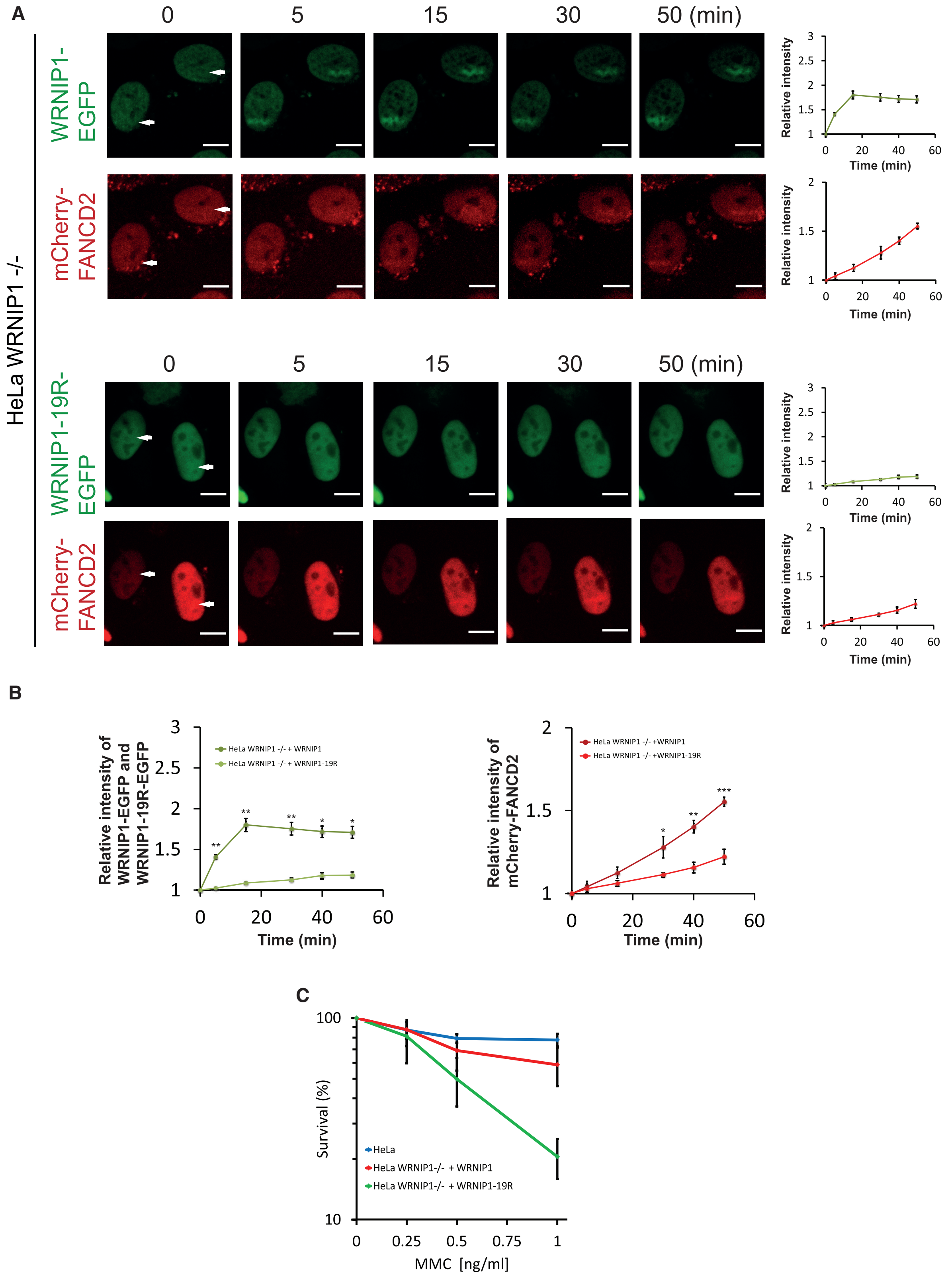

(legend on next page)

revealed that the N33 and D37 residues are well conserved, whereas A45 is not (Figure 6B).

We speculated that the interaction between ubiquitin and WRNIP1's UBZ domain could be important for the ICL repair function of WRNIP1. To investigate this possibility, we decided to create specific point mutations in WRNIP1 to disrupt the interaction. Mutating the zinc-coordinating amino acids (C20, C23, H35, or C39) would abrogate the interaction; however, such mutations could cause larger conformational changes in WRNIP1. Therefore, we instead chose to mutate N33 and D37, individually, to alanines. As expected, mutating these residues has been shown to disrupt the interaction between WRNIP1's UBZ domain and ubiquitin (Suzuki et al., 2016). WRNIP1-EGFP, WRNIP1-N33A-EGFP, and WRNIP1-D37A-EGFP were expressed in HeLa WRNIP1$^{-/-}$ cells at levels similar to that of endogenous WRNIP1 (Figure S6A). We then assessed the ability of the two UBZ mutant proteins to be recruited to ICLs using live-cell imaging. Although WRNIP1-EGFP was strongly recruited to ICLs within minutes of their appearance, both UBZ point mutant proteins were defective in recruitment to ICLs (Figure S6B).

Because the UBZ domain of WRNIP1 is critical for its recruitment to ICLs, we speculated that this domain might consequently be required for WRNIP1 to facilitate loading of the FANCD2/FANCI complex onto DNA. We stably expressed WRNIP1-EGFP or WRNIP1-D37A-EGFP, together with mCherry-FANCD2 in HeLa WRNIP1$^{-/-}$ cells (Figure S6C), and assessed how the various proteins were recruited to ICLs. As expected, WRNIP1-EGFP was recruited robustly and supported strong recruitment of mCherry-FANCD2 (Figures 6C and 6D). Meanwhile, WRNIP1-D37A-EGFP was barely recruited, resulting in poor recruitment of mCherry-FANCD2 (Figures 6C and 6D).

Given the critical role of the UBZ domain for the recruitment of WRNIP1 to ICLs, we hypothesized that this domain consequently might play an important functional role for WRNIP1 in ICL repair. To test this directly, we evaluated the ability of the WRNIP1-N33A-EGFP and WRNIP1-D37A-EGFP proteins to functionally complement HeLa WRNIP1$^{-/-}$ cells. The two proteins were expressed stably at endogenous levels in HeLa WRNIP1$^{-/-}$ cells, and the ability of the resulting cell lines to survive upon introduction of an increasing amount of ICLs was assessed. Although WRNIP1-EGFP fully restored resistance to ICLs, both WRNIP1-N33A-EGFP and WRNIP1-D37A-EGFP were defective (Figure 6E).

These experiments show that two events are critical for the ICL repair function of WRNIP1. First, its ubiquitination is essential, and second, the activity of its UBZ domain is required for repair. However, if the activity of the UBZ domain were affected in the WRNIP1-19R mutant, interpretation of these data would be different. Although none of the point mutations are within the UBZ domain (Figure 4E), it is important to ensure that the full-length protein maintains full ubiquitin binding activity like wild-type WRNIP1. Therefore, we expressed and purified the WRNIP1 and WRNIP1-19R proteins from Sf9 cells (Figure S7) and assessed their abilities to interact with recombinant ubiquitin. Both proteins interacted equally well with ubiquitin, demonstrating that the UBZ domain was not affected by the point mutations in WRNIP1-19R (Figure S6D).

Altogether, mechanistically, both ubiquitination of WRNIP1 and activity of its UBZ domain are required for the ICL repair function of WRNIP1 in human cells.

DISCUSSION

Repair of ICLs entails a series of key events, most of which require specific and timely recruitment of DNA repair factors to the site of damage. In this work, we have biochemically purified and identified several novel ICL repair factors that are recruited to damaged DNA. The function of one of these factors, WRNIP1, is presented.

WRNIP1 Is Recruited to ICLs in Live Cells

We found that WRNIP1 is recruited to ICLs within minutes of their appearance in the genome. Such kinetic of recruitment is relatively quick compared with other DNA repair proteins, indicating an early role in the repair pathway. For instance, the well-characterized FANCD2 protein is detected later in similar experimental settings (Lopez-Martinez et al., 2019), suggesting that WRNIP1 might act before FANCD2. We found that depletion of cellular WRNIP1 leads to a reduction in recruitment of FANCD2 and of its heterodimerization partner, FANCI. Residual recruitment of the FANCD2/FANCI complex was observed upon depletion of WRNIP1, suggesting the presence of alternative mechanisms to support recruitment in the absence of WRNIP1. Perhaps the ICL sensor proteins UHRF1 and UHRF2 provide this activity (Liang et al., 2015; Motnenko et al., 2018; Tian et al., 2015).

Our cell-based assays suggested an interaction between WRNIP1 and the FANCD2/FANCI complex, and using highly purified recombinant proteins, we demonstrated a direct protein-protein interaction with the FANCD2/FANCI complex, as well as monoubiquitinated FANCD2 in complex with FANCI, or Ub-FANCD2/FANCI. Recent work has demonstrated how monoubiquitination of the FANCD2/FANCI complex leads to a substantial conformational change, creating a more stable ring-like structure by which the complex surrounds the DNA (Alcón et al., 2020; Rennie et al., 2020; Tan et al., 2020; Wang et al., 2020). An important part of the conformational change entails rearrangement of the C-terminal Tower domain of FANCD2, which previously was found to be critical for the activity of the FANCD2/FANCI

Figure 5. Ubiquitination of WRNIP1 Is Required for ICL Repair

(A) Live-cell imaging of HeLa-WRNIP1-EGFP, mCherry-FANCD2 cells and of HeLa-WRNIP1-19R-EGFP, mCherry-FANCD2 cells. Cells were treated with TMP, microirradiated at the indicated areas (white arrows), and followed over time. Quantification shows relative intensities of the irradiated areas. 5 cells were quantified for each chart (mean ± SEM). Scale bar, 10 μm.

(B) Combination of quantification data from (A). Recruitment of WRNIP1-EGFP and WRNIP1-19R-EGFP is shown in green and light green, respectively, on the chart on the left. Recruitment of mCherry-FANCD2 in cells expressing WRNIP1-EGFP or WRNIP1-19R-EGFP is shown in red and light red, respectively, on the chart on the right. *$p < 0.05$, **$p < 0.01$, ***$p < 0.001$ (mean ± SEM).

(C) Clonogenic survival assay of HeLa, HeLa WRNIP1$^{-/-}$+WRNIP1-EGFP, and HeLa WRNIP1$^{-/-}$+WRNIP1-19R-EGFP cells (mean ± SD, n = 2).

CellPress
OPEN ACCESS

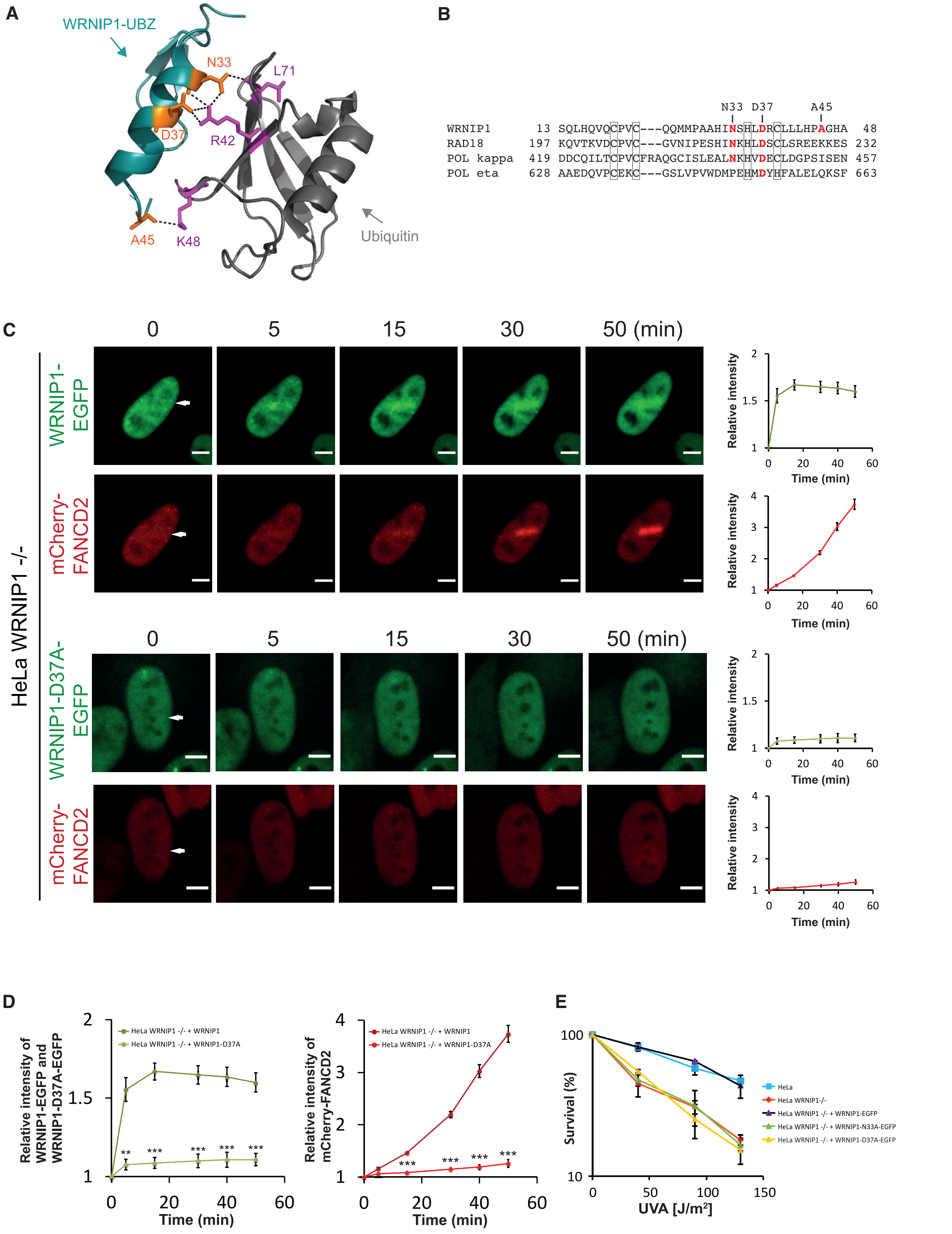

(legend on next page)

complex via its interaction with DNA (Liang et al., 2016). This interaction with DNA is necessary for the complex to become monoubiquitinated and thereby activated (Liang et al., 2016; Sato et al., 2012). However, for the complex to interact with DNA, it first needs to be recruited to DNA, and we found that WRNIP1 triggered this initial step of the pathway.

Altogether, WRNIP1 is an early responder to ICLs that, when recruited, facilitates the subsequent recruitment of the FANCD2/FANCI complex, likely via a direct protein-protein interaction (Figure 7).

WRNIP1 Is Required for Active ICL Repair

Because WRNIP1 facilitates the recruitment of the FANCD2/FANCI complex to ICLs, one would expect cells deficient in WRNIP1 to be sensitive to agents causing ICLs. We found WRNIP1$^{-/-}$ knockout cells to be sensitive to three types of ICLs, namely, those caused by MMC, cisplatin, and TMP/UVA. In addition, cells already deficient in FANCD2 were not further sensitized by WRNIP1 depletion, reinforcing a functional relationship between WRNIP1 and FANCD2 in ICL repair.

The UBZ Domain and Ubiquitination of WRNIP1 Are Required for the ICL Repair Function

WRNIP1 harbors a UBZ domain, which we found to be strictly required for the ICL repair function of WRNIP1. Unconventional amino acid interactions between the UBZ domain and ubiquitin (Suzuki et al., 2016) ensure functional binding, and it is known that this domain can interact with free ubiquitin, as well as with K48 and K63 chains (Crosetto et al., 2008). It will be interesting to determine which ubiquitinated protein or proteins WRNIP1 interacts with during ICL repair (Figure 7). WRNIP1 was previously shown to interact with monoubiquitinated PCNA in yeast (Hishida et al., 2006) and humans (Saugar et al., 2012), an interaction that was later reported to involve ATMIN to augment ATR signaling in response to replication stress (Kanu et al., 2016). WRNIP1 was previously shown to bind DNA directly (Kim et al., 2005; Yoshimura et al., 2009), an activity that could contribute to its recruitment to chromatin.

We also found that ubiquitination of WRNIP1 is required for its ICL repair activity, similar to that of FANCD2 and FANCI. WRNIP1 is reported to functionally interact with RAD18 in chicken cells (Yoshimura et al., 2006), as well as in human cells (Yoshimura et al., 2009). Furthermore, RAD18 is known to be ubiquitinated; thus, WRNIP1 may form contacts to ubiquitinated RAD18 via its UBZ domain. It is also possible that RAD6/RAD18 directly contributes to the ubiquitination of WRNIP1, thereby facilitating repair. It will be important to determine which ubiquitin E3 ligase or ligases act on WRNIP1.

In addition to its role in translesion synthesis, RAD18 was shown to play an active role in HR (Huang et al., 2009). Likewise, WRNIP1 was shown to stabilize RAD51 filaments in response to replication stress, thereby preventing unsolicited resection by MRE11 (Leuzzi et al., 2016). Perhaps this mechanism of resection control involves recruitment of the FANCD2/FANCI complex. We found MRE11 to be a subunit of the purified FANCD2 complex. It is possible that WRNIP1 contributes to stabilization of stalled replication forks as part of the ICL repair by recruiting and stabilizing the FANCD2/FANCI complex at the site of damage.

WRNIP1 Is Important for Several Types of DNA Transactions

DNA synthesis is an important element of ICL repair. WRNIP1 was shown to interact with DNA polymerase delta in yeast (Branzei et al., 2002) and humans (Tsurimoto et al., 2005). We identified three of the four DNA polymerase delta subunits (POLD1, POLD2, and POLD3) as subunits of the purified FANCD2 complex. WRNIP1 was found to stimulate the DNA polymerase activity of polymerase delta *in vitro* by increasing its initiation activity (Tsurimoto et al., 2005). WRNIP1 was also shown to stimulate the FEN1 endonuclease in yeast, thereby positively affecting Okazaki fragment processing (Kim et al., 2005) and agreeing with related findings in humans (Ward et al., 2017). We identified both FEN1 and PRIM2 in our FANCD2 complex. PRIM2 primase is the enzyme synthesizing small RNA primers for Okazaki fragments. Furthermore, WRNIP1 was recently shown to be functionally linked to Primpol via a direct interaction (Yoshimura et al., 2019). Altogether, it is possible that WRNIP1 directly or indirectly promotes DNA synthesis as part of the ICL repair reaction. WRNIP1 shares sequence similarity with the bacterial RuvB motor protein, which drives branch migration during Holiday junction resolution as a part of HR (Yamada et al., 2002). Future studies will reveal whether WRNIP1 also possesses such activities during ICL repair.

Conclusions

We report the identification of WRNIP1 as a new ICL repair factor operating within the FA DNA repair pathway. WRNIP1 is recruited to ICLs quickly after their appearance in the genome in a process requiring both ubiquitination of WRNIP1 and activity of its intrinsic UBZ domain. Mechanistically,

Figure 6. The UBZ Domain of WRNIP1 Is Necessary for Its Recruitment to ICLs

(A) Three potential polar interactions between WRNIP1's UBZ domain and ubiquitin are indicated with dashed lines (GFP-WRNIP1 UBZ/ubiquitin, PDB: 3VHT).

(B) Alignment of UBZ domains from WRNIP1, RAD18, polymerase kappa, and polymerase eta. Amino acids coordinating zinc are shown in black boxes. Amino acids forming contacts to ubiquitin are shown in red.

(C) Live-cell imaging of WRNIP1-EGFP, WRNIP1-D37A-EGFP, and mCherry-FANCD2 in HeLa WRNIP1$^{-/-}$ cells. Cells were treated with TMP, microirradiated at the indicated areas (white arrows), and followed over time. Quantification shows relative intensities of the irradiated areas. 4 cells were quantified for each chart (mean ± SEM). Scale bar, 10 μm.

(D) Combination of quantification data from (C). Recruitment of WRNIP1-EGFP and WRNIP1-D37A-EGFP is shown in green and light green, respectively, on the chart on the left. Recruitment of mCherry-FANCD2 in cells expressing WRNIP1-EGFP or WRNIP1-D37A-EGFP is shown in red and light red, respectively, on the chart on the right. $^{**}p < 0.01$, $^{***}p < 0.001$ (mean ± SEM).

(E) Clonogenic survival assay of HeLa cells and derivative cell lines in response to TMP/UVA. The presented data represents 2 independent experiments with 3 technical repeats each (mean ± SEM, n = 2).

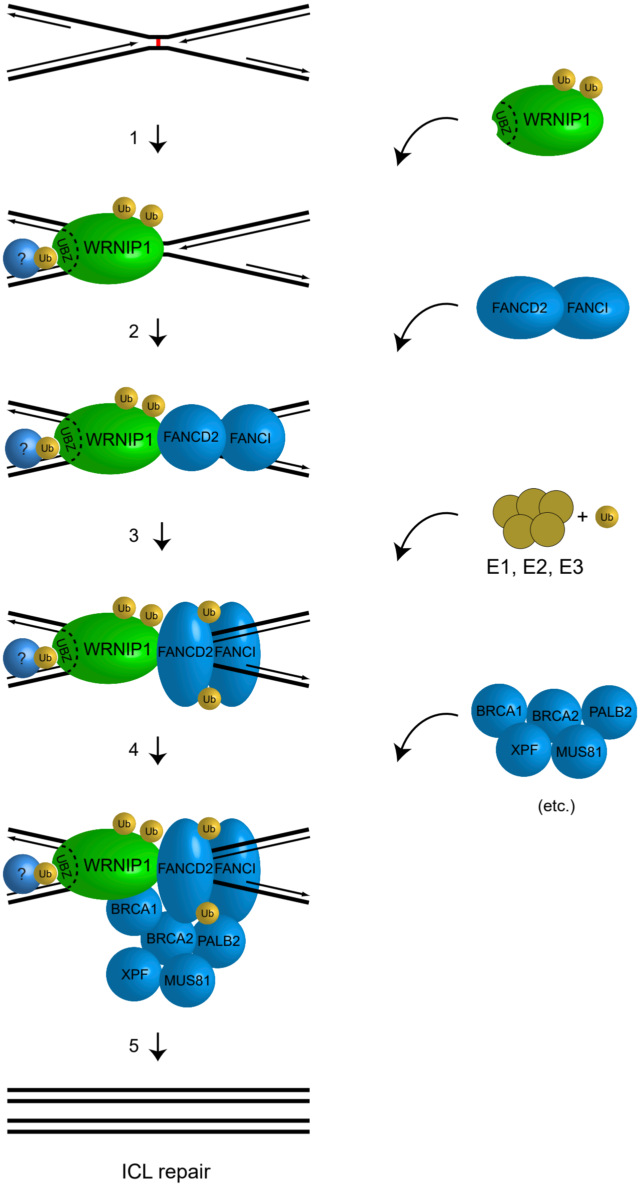

Figure 7. Model Showing How WRNIP1 Functions in ICL Repair
(1) WRNIP1 is recruited to an ICL in the chromatin, potentially assisted by a hypothetical protein that is ubiquitinated. (2) The FANCD2/FANCI complex is recruited, causing a conformational change upon interaction with DNA. (3) The FANCD2/FANCI complex is monoubiquitinated, causing another conformational change that leads to its ring-like structure encircling the DNA helix. (4) Additional ICL repair factors are recruited. (5) The ICL is repaired.

recruitment of WRNIP1 facilitates subsequent recruitment of the FANCD2/FANCI complex, in turn initiating DNA repair. Importantly, this work also reports the identification of several additional new ICL repair factors. A few of these are discussed in the paper; however, most of these factors are presented as a list of subunits identified in the purified complex. We hope that reporting this full list of subunits will provide a rich source for other investigators to study DNA repair in humans and likely other species.

STAR★METHODS

Detailed methods are provided in the online version of this paper and include the following:

- KEY RESOURCES TABLE
- RESOURCE AVAILABILITY
  - Lead Contact
  - Materials Availability
  - Data and Code Availability
- EXPERIMENTAL MODEL
  - Cell lines
- METHOD DETAILS
  - Plasmids and transfection
  - Immunoblot analysis
  - ICL induction with TMP
  - Clonogenic survival assay
  - Protein purification
  - Mass Spectrometric Analysis
  - Protein-protein interaction assay
  - Ubiquitin binding assay
  - Live-cell microscopy
- QUANTIFICATION AND STATISTICAL ANALYSIS

SUPPLEMENTAL INFORMATION

Supplemental Information can be found online at https://doi.org/10.1016/j.celrep.2020.107850.

ACKNOWLEDGMENTS

We thank members of the Cohn laboratory for helpful discussions and comments. We thank Luke Lavis (Janelia Research Campus) for providing the JF549 Halo ligand dye. We are grateful to staff at the Micron microscopy facility for excellent help and support. We thank the Dunn School of Pathology Proteomics Facility for some of the mass spectrometry experiments. This work was supported by grants UF100717 and UF150651 from the Royal Society (to M.A.C.), grant 153/092 from The Fell Fund (to M.A.C.), grants OCRC 0213-MC and CRUKDF 0715-MC from the Oxford Cancer Research Centre (OCRC)/Cancer Research UK (CR-UK) (to M.A.C.), a grant from the Medical Research Fund (to M.A.C.), grant MR/N021002/1 from the Medical Research Council (MRC) (to M.A.C.), Wellcome Trust Senior Research Fellowship 210640/Z/18/Z (to M.A.C.), a Clarendon scholarship from the Clarendon Fund (to K.Y.), a Cyril and Phillis Long Scholarship from The Queen's College, Oxford (to K.Y.), the Taiwanese Government (to C.-C.L.), a Goodger scholarship (to C.-C.L.), and a studentship from the Biotechnology and Biological Sciences Research Council (BBSRC) (to A.B.).

AUTHOR CONTRIBUTIONS

A.S., D.Y., A.B., K.Y., M.K., C.-C.L., A.H., R.W., S.P.G., and M.A.C. designed and analyzed the experiments; A.S., D.Y., A.B., K.Y., M.K., C.-C.L., A.H., and R.W. performed the experiments; A.S., D.Y., A.B., K.Y., M.K., C.-C.L., A.H., R.W., S.P.G., and M.A.C. prepared the manuscript.

DECLARATION OF INTERESTS

The authors declare no competing interests.

Received: September 9, 2019
Revised: January 15, 2020
Accepted: June 11, 2020
Published: July 7, 2020

REFERENCES

Alcón, P., Shakeel, S., Chen, Z.A., Rappsilber, J., Patel, K.J., and Passmore, L.A. (2020). FANCD2-FANCI is a clamp stabilized on DNA by monoubiquitination of FANCD2 during DNA repair. Nat. Struct. Mol. Biol. *27*, 240–248.

Bish, R.A., and Myers, M.P. (2007). Werner helicase-interacting protein 1 binds polyubiquitin via its zinc finger domain. J. Biol. Chem. *282*, 23184–23193.

Bish, R.A., Fregoso, O.I., Piccini, A., and Myers, M.P. (2008). Conjugation of complex polyubiquitin chains to WRNIP1. J. Proteome Res. *7*, 3481–3489.

Branzei, D., Seki, M., Onoda, F., and Enomoto, T. (2002). The product of Saccharomyces cerevisiae WHIP/MGS1, a gene related to replication factor C genes, interacts functionally with DNA polymerase delta. Mol. Genet. Genomics *268*, 371–386.

Cohn, M.A., Kowal, P., Yang, K., Haas, W., Huang, T.T., Gygi, S.P., and D'Andrea, A.D. (2007). A UAF1-containing multisubunit protein complex regulates the Fanconi anemia pathway. Mol. Cell *28*, 786–797.

Crosetto, N., Bienko, M., Hibbert, R.G., Perica, T., Ambrogio, C., Kensche, T., Hofmann, K., Sixma, T.K., and Dikic, I. (2008). Human Wrnip1 is localized in replication factories in a ubiquitin-binding zinc finger-dependent manner. J. Biol. Chem. *283*, 35173–35185.

Dong, H., Nebert, D.W., Bruford, E.A., Thompson, D.C., Joenje, H., and Vasiliou, V. (2015). Update of the human and mouse Fanconi anemia genes. Hum. Genomics *9*, 32.

Eng, J.K., McCormack, A.L., and Yates, J.R. (1994). An approach to correlate tandem mass spectral data of peptides with amino acid sequences in a protein database. J. Am. Soc. Mass Spectrom. *5*, 976–989.

Fagerlie, S.R., and Bagby, G.C. (2006). Immune defects in Fanconi anemia. Crit. Rev. Immunol. *26*, 81–96.

Giri, N., Batista, D.L., Alter, B.P., and Stratakis, C.A. (2007). Endocrine abnormalities in patients with Fanconi anemia. J. Clin. Endocrinol. Metab. *92*, 2624–2631.

Haas, W., Faherty, B.K., Gerber, S.A., Elias, J.E., Beausoleil, S.A., Bakalarski, C.E., Li, X., Villén, J., and Gygi, S.P. (2006). Optimization and use of peptide mass measurement accuracy in shotgun proteomics. Mol. Cell. Proteomics *5*, 1326–1337.

Hishida, T., Iwasaki, H., Ohno, T., Morishita, T., and Shinagawa, H. (2001). A yeast gene, MGS1, encoding a DNA-dependent AAA(+) ATPase is required to maintain genome stability. Proc. Natl. Acad. Sci. USA *98*, 8283–8289.

Hishida, T., Ohya, T., Kubota, Y., Kamada, Y., and Shinagawa, H. (2006). Functional and physical interaction of yeast Mgs1 with PCNA: impact on RAD6-dependent DNA damage tolerance. Mol. Cell Biol. *26*, 5509–5517. https://doi.org/10.1128/MCB.00307-06.

Huang, J., Huen, M.S., Kim, H., Leung, C.C., Glover, J.N., Yu, X., and Chen, J. (2009). RAD18 transmits DNA damage signalling to elicit homologous recombination repair. Nat. Cell Biol. *11*, 592–603.

Kanamori, M., Seki, M., Yoshimura, A., Tsurimoto, T., Tada, S., and Enomoto, T. (2011). Werner interacting protein 1 promotes binding of Werner protein to template-primer DNA. Biol. Pharm. Bull. *34*, 1314–1318.

Kanu, N., Zhang, T., Burrell, R.A., Chakraborty, A., Cronshaw, J., DaCosta, C., Grönroos, E., Pemberton, H.N., Anderton, E., Gonzalez, L., et al. (2016). RAD18, WRNIP1 and ATMIN promote ATM signalling in response to replication stress. Oncogene *35*, 4009–4019.

Kawabe, Y., Branzei, D., Hayashi, T., Suzuki, H., Masuko, T., Onoda, F., Heo, S.J., Ikeda, H., Shimamoto, A., Furuichi, Y., et al. (2001). A novel protein interacts with the Werner's syndrome gene product physically and functionally. J. Biol. Chem. *276*, 20364–20369.

Kawabe, Y., Seki, M., Yoshimura, A., Nishino, K., Hayashi, T., Takeuchi, T., Iguchi, S., Kusa, Y., Ohtsuki, M., Tsuyama, T., et al. (2006). Analyses of the interaction of WRNIP1 with Werner syndrome protein (WRN) *in vitro* and in the cell. DNA Repair (Amst.) *5*, 816–828.

Kim, J.H., Kang, Y.H., Kang, H.J., Kim, D.H., Ryu, G.H., Kang, M.J., and Seo, Y.S. (2005). *In vivo* and *in vitro* studies of Mgs1 suggest a link between genome instability and Okazaki fragment processing. Nucleic Acids Res. *33*, 6137–6150.

Lau, I.F., Filipe, S.R., Søballe, B., Økstad, O.A., Barre, F.X., and Sherratt, D.J. (2003). Spatial and temporal organization of replicating Escherichia coli chromosomes. Mol. Microbiol. *49*, 731–743.

Leuzzi, G., Marabitti, V., Pichierri, P., and Franchitto, A. (2016). WRNIP1 protects stalled forks from degradation and promotes fork restart after replication stress. EMBO J. *35*, 1437–1451.

Liang, C.C., Zhan, B., Yoshikawa, Y., Haas, W., Gygi, S.P., and Cohn, M.A. (2015). UHRF1 is a sensor for DNA interstrand crosslinks and recruits FANCD2 to initiate the Fanconi anemia pathway. Cell Rep. *10*, 1947–1956.

Liang, C.C., Li, Z., Lopez-Martinez, D., Nicholson, W.V., Vénien-Bryan, C., and Cohn, M.A. (2016). The FANCD2-FANCI complex is recruited to DNA interstrand crosslinks before monoubiquitination of FANCD2. Nat. Commun. *7*, 12124.

Lopez-Martinez, D., Liang, C.C., and Cohn, M.A. (2016). Cellular response to DNA interstrand crosslinks: the Fanconi anemia pathway. Cell. Mol. Life Sci. *73*, 3097–3114.

Lopez-Martinez, D., Kupculak, M., Yang, D., Yoshikawa, Y., Liang, C.-C., Wu, R., Gygi, S.P., and Cohn, M.A. (2019). Phosphorylation of $FANCD_2$ Inhibits the $FANCD_2$/FANCI Complex and Suppresses the Fanconi Anemia Pathway in the Absence of DNA Damage. Cell Rep. *27*, 2990–3005.

Lossaint, G., Larroque, M., Ribeyre, C., Bec, N., Larroque, C., Décaillet, C., Gari, K., and Constantinou, A. (2013). FANCD2 binds MCM proteins and controls replisome function upon activation of s phase checkpoint signaling. Mol. Cell *51*, 678–690.

Marabitti, V., Lillo, G., Malacaria, E., Palermo, V., Pichierri, P., and Franchitto, A. (2020). Checkpoint Defects Elicit a WRNIP1-Mediated Response to Counteract R-Loop-Associated Genomic Instability. Cancers (Basel) *12*, 389.

Motnenko, A., Liang, C.C., Yang, D., Lopez-Martinez, D., Yoshikawa, Y., Zhan, B., Ward, K.E., Tian, J., Haas, W., Spingardi, P., et al. (2018). Identification of UHRF2 as a novel DNA interstrand crosslink sensor protein. PLoS Genet. *14*, e1007643.

Nakatani, Y., and Ogryzko, V. (2003). Immunoaffinity purification of mammalian protein complexes. Methods Enzymol. *370*, 430–444.

Niraj, J., Färkkilä, A., and D'Andrea, A.D. (2019). The Fanconi Anemia Pathway in Cancer. Annu. Rev. Cancer Biol. *3*, 457–478.

Nomura, H., Yoshimura, A., Edo, T., Kanno, S., Tada, S., Seki, M., Yasui, A., and Enomoto, T. (2012). WRNIP1 accumulates at laser light irradiated sites rapidly via its ubiquitin-binding zinc finger domain and independently from its ATPase domain. Biochem. Biophys. Res. Commun. *417*, 1145–1150.

Pladevall-Morera, D., Munk, S., Ingham, A., Garribba, L., Albers, E., Liu, Y., Olsen, J.V., and Lopez-Contreras, A.J. (2019). Proteomic characterization of chromosomal common fragile site (CFS)-associated proteins uncovers ATRX as a regulator of CFS stability. Nucleic Acids Res. *47*, 8004–8018.

Porebski, B., Wild, S., Kummer, S., Scaglione, S., Gaillard, P.L., and Gari, K. (2019). WRNIP1 Protects Reversed DNA Replication Forks from SLX4-Dependent Nucleolytic Cleavage. iScience *21*, 31–41.

Rennie, M.L., Lemonidis, K., Arkinson, C., Chaugule, V.K., Clarke, M., Streetley, J., Spagnolo, L., and Walden, H. (2020). Differential functions of FANCI and FANCD2 ubiquitination stabilize ID2 complex on DNA. EMBO Rep. Published online June 8, 2020. https://doi.org/10.15252/embr.202050133.

Sato, K., Toda, K., Ishiai, M., Takata, M., and Kurumizaka, H. (2012). DNA robustly stimulates FANCD2 monoubiquitylation in the complex with FANCI. Nucleic Acids Res. *40*, 4553–4561.

Saugar, I., Parker, J.L., Zhao, S., and Ulrich, H.D. (2012). The genome maintenance factor Mgs1 is targeted to sites of replication stress by ubiquitylated PCNA. Nucleic Acids Res. *40*, 245–257.

Sherratt, D.J., Søballe, B., Barre, F.X., Filipe, S., Lau, I., Massey, T., and Yates, J. (2004). Recombination and chromosome segregation. Philos. Trans. R. Soc. Lond. B Biol. Sci. *359*, 61–69.

CellPress
OPEN ACCESS

Suzuki, N., Rohaim, A., Kato, R., Dikic, I., Wakatsuki, S., and Kawasaki, M. (2016). A novel mode of ubiquitin recognition by the ubiquitin-binding zinc finger domain of WRNIP1. FEBS J. *283*, 2004–2017.

Tan, W., van Twest, S., Leis, A., Bythell-Douglas, R., Murphy, V.J., Sharp, M., Parker, M.W., Crismani, W., and Deans, A.J. (2020). Monoubiquitination by the human Fanconi anemia core complex clamps FANCI:FANCD2 on DNA in filamentous arrays. eLife *9*, e54128.

Tian, Y., Paramasivam, M., Ghosal, G., Chen, D., Shen, X., Huang, Y., Akhter, S., Legerski, R., Chen, J., Seidman, M.M., et al. (2015). UHRF1 contributes to DNA damage repair as a lesion recognition factor and nuclease scaffold. Cell Rep. *10*, 1957–1966.

Tsurimoto, T., Shinozaki, A., Yano, M., Seki, M., and Enomoto, T. (2005). Human Werner helicase interacting protein 1 (WRNIP1) functions as a novel modulator for DNA polymerase delta. Genes Cells *10*, 13–22.

Vijeh Motlagh, N.D., Seki, M., Branzei, D., and Enomoto, T. (2006). Mgs1 and Rad18/Rad5/Mms2 are required for survival of Saccharomyces cerevisiae mutants with novel temperature/cold sensitive alleles of the DNA polymerase delta subunit, Pol31. DNA Repair (Amst.) *5*, 1459–1474.

Wang, R., Wang, S., Dhar, A., Peralta, C., and Pavletich, N.P. (2020). DNA clamp function of the monoubiquitinated Fanconi anaemia ID complex. Nature *580*, 278–282.

Ward, T.A., McHugh, P.J., and Durant, S.T. (2017). Small molecule inhibitors uncover synthetic genetic interactions of human flap endonuclease 1 (FEN1) with DNA damage response genes. PLoS ONE *12*, e0179278.

Yamada, K., Miyata, T., Tsuchiya, D., Oyama, T., Fujiwara, Y., Ohnishi, T., Iwasaki, H., Shinagawa, H., Ariyoshi, M., Mayanagi, K., and Morikawa, K. (2002). Crystal structure of the RuvA-RuvB complex: a structural basis for the Holliday junction migrating motor machinery. Mol. Cell *10*, 671–681.

Yoshimura, A., Seki, M., Hayashi, T., Kusa, Y., Tada, S., Ishii, Y., and Enomoto, T. (2006). Functional relationships between Rad18 and WRNIP1 in vertebrate cells. Biol. Pharm. Bull. *29*, 2192–2196.

Yoshimura, A., Seki, M., Kanamori, M., Tateishi, S., Tsurimoto, T., Tada, S., and Enomoto, T. (2009). Physical and functional interaction between WRNIP1 and RAD18. Genes Genet. Syst. *84*, 171–178.

Yoshimura, A., Kobayashi, Y., Tada, S., Seki, M., and Enomoto, T. (2014). WRNIP1 functions upstream of DNA polymerase η in the UV-induced DNA damage response. Biochem. Biophys. Res. Commun. *452*, 48–52.

Yoshimura, A., Oikawa, M., Jinbo, H., Hasegawa, Y., Enomoto, T., and Seki, M. (2019). WRNIP1 Controls the Amount of PrimPol. Biol. Pharm. Bull. *42*, 764–769.

STAR★METHODS

KEY RESOURCES TABLE

REAGENT or RESOURCE	SOURCE	IDENTIFIER
Antibodies		
Mouse monoclonal anti-FANCD2 (FY17)	Santa Cruz Biotechnology	Cat# sc-20022; RRID: AB_2278211
Mouse monoclonal anti-α-Tubulin (DM1A)	Merck-Millipore	Cat# 05-829; RRID: AB_310035
Mouse monoclonal anti-WRNIP1 (WHIP A8)	Santa Cruz Biotechnology	Cat# Sc-376438; RRID: AB_11149006
Mouse monoclonal anti-FLAG (M5)	Sigma-Aldrich	Cat# F4042; RRID: AB_439686
Mouse monoclonal anti-HA	Roche	Cat# 11583816001; RRID: AB_514505
Mouse monoclonal anti-Ubiquitin (FK2)	Merck-Millipore	Cat# ST1200; RRID: AB_2043482
Mouse monoclonal anti-poly-Histidine	Sigma-Aldrich	Cat# H1029; RRID: AB_260015
Anti-IL-2 Rα Antibody, clone 7G7/B6	Merck-Millipore	Cat# 05-170; RRID: AB_309642
Anti-Mouse (Horseradish Peroxidase conjugated)	GE Healthcare	NA9310V
Bacterial and Virus Strains		
Top10	Thermo Fisher	Cat# 404010
Chemicals, Peptides, and Recombinant Proteins		
FuGENE6	Promega	Cat# E2691
FLAG-HA-FANCD2	Liang et al., 2016	N/A
His-Strep(II)-WRNIP1	This paper	N/A
His-MBP	Motnenko et al., 2018	N/A
Protein A Sepharose Cl-4B	Thermo Fisher	Cat# GE17-0963-02
Dynabeads Goat Anti-Mouse IgG	Thermo Fisher	Cat# 11033
Anti-FLAG M2 agarose resin	Sigma-Aldrich	Cat# A2220
Ni^{2}–NTA agarose resin	QIAGEN	Cat# 30210
Trioxsalen (TMP)	Sigma-Aldrich	Cat# 6137
Mitomycin C from *Streptomyces caespitosus*	Sigma-Aldrich	Cat# M4287
Benzonase	Sigma-Aldrich	Cat# E1014
Critical Commercial Assays		
Sequencing	Source BioScience	N/A
Deposited Data		
Mass spectrometry	This paper, and Mendeley Data	Table S1; https://doi.org/10.17632/z7nx93j39j.1
Experimental Models: Cell Lines		
HeLa FANCD2−/−	Liang et al., 2016	N/A
HeLa Flp-in T-Rex cells	Thermo fisher	N/A
HeLa Flp-in WRNIP1 −/−	This paper	N/A
HeLa Flp-in WRNIP1-EGFP + mCherry-FANCD2	This paper	N/A
HeLa Flp-in WRNIP1 −/− + WRNIP1-EGFP	This paper	N/A
HeLa FANCD2 −/− WRNIP1−/−	This paper	N/A
HeLa expressing mCherry-UHRF1 and EGFP-FANCD2	This paper	N/A
HeLa expressing mCherry-UHRF1 and EGFP-FANCI	This paper	N/A
HeLa WRNIP1 −/− expressing WRNIP1-EGFP and mCherry-UHRF1	This paper	N/A
HeLa WRNIP1 −/− expressing WRNIP1-19R-EGFP and mCherry-UHRF1	This paper	N/A
HeLa expressing Flag-HA-tagged FANCD2	Liang et al., 2016	N/A
HeLa S3 expressing Flag-HA-WRNIP1	This paper	N/A
HeLa Flp-in WRNIP1 −/− +WRNIP1-N33A-EGFP	This paper	N/A

(*Continued on next page*)

Continued

REAGENT or RESOURCE	SOURCE	IDENTIFIER
HeLa Flp-in WRNIP1 −/− +WRNIP1-D37A-EGFP	This paper	N/A
HeLa Flp-in WRNIP1 −/− +WRNIP1-K301R-EGFP	This paper	N/A
HeLa Flp-in WRNIP1 −/− +WRNIP1-K310R-EGFP	This paper	N/A
HeLa Flp-in WRNIP1 −/− +WRNIP1-K335R-EGFP	This paper	N/A
HeLa Flp-in WRNIP1 −/− +WRNIP1-K633R-EGFP	This paper	N/A
HeLa Flp-in WRNIP1 −/− +WRNIP1-K636R-EGFP	This paper	N/A
Oligonucleotides		
Primer used to create the Flp-in T-REx HeLa WRNIP1-EGFP cell line (forward primer): 5′-AGTCGGATCCACCATGGAGGTGAGCGGGCCGGAAG-3′	This paper	N/A
Primer used to create the Flp-in T-REx HeLa WRNIP1-EGFP cell line (reverse primer): 5′-AGTCGTCGACGCACCTCCTCTGCTTGAAGAAATCTACC-3′	This paper	N/A
Primer used to create plasmid disrupting the *WRNIP1* gene by CRISPR (forward primer): 5′-CACCGGCGAGTTGATGTGCGCGGC-3′	This paper	N/A
Primer used to create plasmid disrupting the *WRNIP1* gene by CRISPR (reverse primer): 5′-AAACGCCGCGCACATCAACTCGCC-3′	This paper	N/A
Recombinant DNA		
pOZ-N	Nakatani and Ogryzko, 2003	N/A
pBlueScript II SK (+)	Addgene	Cat# 10359-016
pcDNA5-FRT/TO-EGFP	Addgene	Cat# 212205
pOZ-Puro-mCherry	Liang et al., 2016	N/A
pFB-FLAG-HA	Liang et al., 2016	N/A
pFastBac1	Thermo-Fisher	Cat# 10360014
pSpCas9(BB)-2A-Puro (PX459)	Addgene	Cat# 48139
pOG44	Thermo-Fisher	Cat# V600520
Software and Algorithms		
Volocity	Quorum Technologies	N/A
Fiji	Fiji.sc	N/A
PyMOL	Pymol.org	N/A
SEQUEST algorithm	Eng et al., 1994	N/A
Other		
MS/MS analysis of purified FANCD2 complex (hybrid linear ion trap/FT-ICR mass spectrometer LTQ-FT, Thermo Electron)	Harvard Medical School, Boston	N/A
MS/MS analysis of purified ubiquitinated WRNIP1	Dunn School of Pathology Proteomics Facility	N/A
PE Ultraview Spinning Disk Microscope	Micron Facility, University of Oxford	N/A
Spectrolinker XL-1500 (365 nm)	Department of Biochemistry, University of Oxford	N/A

RESOURCE AVAILABILITY

Lead Contact

Further information and requests for resources and reagents should be directed to and will be fulfilled by the Lead Contact, Martin A. Cohn (martin.cohn@bioch.ox.ac.uk).

Materials Availability

Materials will be provided upon request to Lead Contact.

Data and Code Availability

Original mass spectrometry data relating to Figure 1 and Table S1 have been deposited to Mendeley Data: https://dx.doi.org/10.17632/z7nx93j39j.1.

EXPERIMENTAL MODEL

Cell lines

HeLa, HeLa S3, HeLa Flp-in T-REx and Phoenix A cells in dishes were grown in DMEM (D5796, Sigma) supplemented with 2.5%–10% fetal bovine serum.

METHOD DETAILS

Plasmids and transfection

EGFP-fused WRNIP1 cDNA was expressed using the pcDNA5-FRT/TO-EGFP plasmid, under a Doxycycline-regulated promotor. WRNIP1 deficient cell lines were obtained using the CRISPR-Cas9 gene editing technology and the targeting sequence used in the sgRNA was: 5′-TGCGAGTTGATGTGCGCGGC-3′, the pX459 (Addgene #48139) was used as a vector for the sgRNA sequence. Cells were transfected with 1 μg of the resulting pX459 plasmid and selected with 4 μg/ml puromycin after 24 h. After another 24 h, cells were plated at low density and clones were picked after 2 weeks. Clones were analyzed using immunoblot analysis. Flag-HA-WRNIP1 was expressed in HeLa cells using the pOZ-N vector.

Immunoblot analysis

For analysis of whole cell lysate, cells were grown in 9 cm plates in an incubator overnight, then scraped off with a plastic spatula. The medium with the scraped off cells was transferred to a 15 mL Falcon tube and spun down (1,000 rpm, 5 min), then resuspended in 1 mL of phosphate-buffered saline (PBS) buffer, transferred into an Eppendorf tube and spun down (4,000 rpm, 2 min, 4°C). PBS was aspirated, the tube spun down again and the remains of the supernatant was aspirated. The volume of the pellet was estimated and an equal volume of benzonase buffer (20 mM Tris (pH 8.0), 10% glycerol, 2 mM $MgCl_2$, 1% Triton X-100) with Benzonase (12.5 units/ml) was added. The sample was vortexed then put on ice for 10 min. Thereafter an equal volume of 2% sodium dodecyl sulfate (SDS) (twice the volume of the pellet) was added and the sample was vortexed and incubated at 70°C for 2 min. The amount of protein in each lysate sample was measured and samples for SDS-PAGE were prepared with the concentration of 1 μg/μl. Antibodies were used as follows, anti-WRNIP1 (WHIP A8, sc-376438, Santa Cruz Biotechnology), 1:3,000 or 1:10,000 dilution depending on the experiment; anti-FANCD2 (sc-20022, Santa Cruz Biotechnology), 1:100 dilution; anti-Flag (M5, F4042, Sigma-Aldrich), 1:1,000 dilution, anti-HA (12CA5, 11583816001, Roche) 1:1,000 dilution; anti-Ubiquitin (FK2, ST1200, Merck-Millipore), 1:400 dilution; anti-α-Tubulin (5829, Millipore), 1:2,000 dilution; and anti-poly-Histidine (H1029, Sigma-Aldrich) 1:1,000 dilution.

ICL induction with TMP

Cells were incubated with 2 μg/ml TMP for 30 min at 37°C and then irradiated with 50 mJ/cm^2 UVA (365 nm) in a Spectrolinker XL-1500.

Clonogenic survival assay

Cells (250–4,000) were plated in six-well plates and treated as indicated on the next day. Colony formation was scored after 10–14 days using 1% (w/v) crystal violet in methanol.

Protein purification

Purification of FANCD2-containing complexes. HeLa S3 cells stably expressing Flag-HA-FANCD2 were cultured in Joklik's medium in suspension supplemented with non-essential amino acids, L-glutamine and Penicillin-Streptomycin, and treated with 160 ng/ml mitomycin C overnight. The cells were harvested by centrifugation at 3,000 rpm for 5 min. The cell pellet was resuspended in cold PBS, and centrifuged at 3,000 rpm for 5 min. The cell pellet was then resuspended with hypotonic buffer (10 mM KCl, 10 mM Tris-HCl (pH 7.3), 2 mM 2-mercaptoethanol, 0.2 mM PMSF and 1.5 mM $MgCl_2$), and incubated on ice for 10 min. Cells were ruptured using a Dounce homogenizer, followed by centrifugation at 3,900 rpm for 15 min at 4°C to separate the cytoplasmic fraction and nuclei. The nuclei pellet was resuspended in 0.5 pellet volume of low salt nuclear extraction buffer (50 mM KCl, 20 mM Tris-HCl (pH 7.3), 1.5 mM $MgCl_2$, 0.2 mM EDTA, 2 mM 2-mercaptoethanol, 0.2 mM PMSF and 25% glycerol) supplemented with 75 units/ml Benzonase (Merck Millipore, 70664), and incubated on ice for 30 min. Formaldehyde was added to a final concentration of 1%, and the mixture was incubated at room temperature for 3 min followed by quenching using glycine. The sample was centrifuged at 4,000 rpm for 10 min, and washed with cold PBS three times. The nuclei pellet was resuspended with an equal pellet volume of high salt nuclear extraction buffer (1 M KCl, 20 mM Tris-HCl (pH 7.3), 1.5 mM $MgCl_2$, 0.2 mM EDTA, 1% Tween-20, 0.15% SDS, 2 mM

2-mercaptoethanol, 0.2 mM PMSF and 25% glycerol), and sonicated with 4 W and 1 s on/off interval for 10 min on ice. The mixture was incubated on ice for 30 min, and drop-wise diluted 5-fold to 0.03% SDS, 1% Tween-20 and 0.3M NaCl. The sample was centrifuged at 13,000 rpm for 20 min at 4°C and the supernatant was collected as nuclear extract. Equilibrated M2 anti-FLAG agarose (Sigma) was added to the nuclear extract, and incubated in the cold room for 2 h with constant rotation. The M2 anti-FLAG agarose was washed with a buffer containing 100 mM KCl, 20 mM Tris-HCl (pH 8.0), 0.2 mM EDTA, 0.1% Tween-20, 5 mM $MgCl_2$, 2 mM 2-mercaptoethanol, 0.2 mM PMSF and 10% glycerol, and eluted with the same buffer supplemented with 0.5 mg/ml FLAG peptide. Equilibrated anti-HA Sepharose was added to the FLAG eluate, and incubated in the cold room for 2 h with constant rotation. The anti-HA Sepharose was washed with buffer containing 100 mM Tris-HCl (pH 6.8), 0.2 mM EDTA, 100 mM KCl, 0.1% Tween-20, 2 mM 2-mercaptoethanol, 0.2 mM PMSF and 10% glycerol, and eluted with the same buffer containing 0.5 mg/ml HA peptide. The purified FANCD2-containing complexes were precipitated with 10% TCA. The precipitated pellet was washed with 100% acetone and air-dried before SDS-PAGE/Silver stain or MS/MS analysis.

Purification of Flag-HA-WRNIP1 from HeLa S3 cells for MS/MS analysis. HeLa S3 cells stably expressing Flag-HA-WRNIP1 were split into three 15 cm dishes with approximately 70% confluency. All three dishes were left in 37°C incubator overnight. The next day the cells were harvested and centrifuged at 4,000 rpm for 2 min. The pellet was resuspended in 250 μL of Buffer A (20 mM Tris-HCl (pH 8.0), 0.5% Triton X-100, 10% glycerol, 50 unit/ml Benzonase, 2 mM β-mercaptoethanol, 0.2 mM PMSF (phenylmethylsulfonyl fluoride)) and left on ice for 10 min. 2.5 mL (10x pellet volume) of Buffer B (20 mM Tris-HCl (pH 8.0), 0.5% Triton X-100, 10% glycerol, 200 mM KCl, 2 mM β-mercaptoethanol, 0.2 mM PMSF) was mixed into the sample, and the falcon tube was incubated on ice for 10 min. The solution was spun down at 18,000 rpm for 10 min at 4°C. The supernatant was transferred to new tubes and spun again (18,000 rpm for 10 min at 4°C), before it was collected. 100 μL of M2 beads (α-FLAG) 50% slurry (Sigma-Aldrich) was added, and the falcon tube was incubated for 2 h under gentle rotation (4°C). The lysate/beads slurry was transferred to an empty chromatography column (10 mL volume, equilibrated with 1 mL of Buffer B beforehand) and washed with 5 mL of Buffer B. The column was closed, and 50 μL of elution buffer was added (0.5 μg/μl Flag peptide in buffer B). The lysate/beads slurry was incubated with Flag peptide in the cold room for 1.5 h (mixed every 15 min). The eluate was collected and the elution step was repeated (the same volume of the Flag peptide solution was added, and incubated for 1.5 h), the eluate from the second elution was consolidated with the eluate from the first elution. The samples were then analyzed using SDS-PAGE gel followed by Coomassie blue staining or immunoblot analysis (α-WRNIP1, α-HA and α-Ubiquitin (FK2)). Flag-HA-WRNIP1 analyzed by MS/MS was first precipitated with 10% trichloroacetic acid (TCA) (75 μL Flag eluate plus 75 μL 20% TCA). The sample was incubated on ice for 4 h, and then centrifuged (13,000 rpm, 30 min, 4°C). Supernatant was removed, and the pellet was washed with 800 μL ice cold acetone. The sample was centrifuged again (13,000 rpm, 30 min, 4°C) and the pellet was dried in air for 10 min and resuspended in 10 μL of LDS loading buffer. The sample was analyzed by SDS-PAGE (9%) followed by Coomassie blue staining. The upper form of WRNIP1 was carefully cut out of the gel using a scalpel and provided to the Dunn School of Pathology Proteomics Facility for MS/MS analysis.

Mass Spectrometric Analysis

Initial identification of subunits of the FANCD2 complexes was performed as follows. Flag-HA-FANCD2 purified from HeLa cells was reduced with DTT, cysteine residues were derivatized with iodoacetamide, and the proteins were digested with trypsin. The generated peptide mixtures were subjected to LC-MS/MS using a hybrid linear ion trap/FT-ICR mass spectrometer (LTQ FT, Thermo Electron) essentially as described previously (Haas et al., 2006). MS/MS spectra were assigned by searching them with the SEQUEST algorithm (Eng et al., 1994) against the human International Protein Index sequence database. Analysis of ubiquitinated WRNIP1 was performed by the Dunn School of Pathology Proteomics Facility.

Protein-protein interaction assay

0.5 μg of Flag-HA-FANCD2 and 0.5 μg of His-Strep(II)-WRNIP1 or 0.5 μg of His-MBP were combined in an Eppendorf tube. For the binding assay with the Ub-FANCD2/FANCI complex, Flag-HA-FANCD2/His-FANCI was first monoubiquitinated as previously described (Lopez-Martinez et al., 2019). After the ubiquitination reaction, 3 μL of benzonase (250U/μl) was added followed by 30 min incubation at room temperature. For the binding assay, FANCD2, WRNIP1 or MBP, 1 μL BSA (150 mg/ml) and binding buffer (150 mM KCl, 20 mM Tris-HCl (pH 8.0), 0.2 mM PMSF, 2 mM β-mercapthoethanol) were added to a final volume of 10 μl. The sample was mixed by vortexing and spun quickly. 1.5 μL of the input sample was taken from each Eppendorf for immunoblot analysis. The sample was incubated at 30°C for 1 h. After the incubation the binding buffer was supplemented with 0.1% of Tween-20 (v/v). α-HA Sepharose beads were washed two times in the Tween-20 supplemented binding buffer (resuspended and spun down at 2,000 rpm for 5 min). 10 μL of α-HA bead 50% slurry was added to each Eppendorf tube. The samples were incubated for half an hour with slow rotation in the cold room. Afterwards, the samples were transported into emptry 3 mL chromatography columns and washed with 3 mL of the binding buffer. Thereafter proteins were eluted with 30 μL glycine buffer (0.1 M glycine (pH 2.5)). The column was centrifuged at 1,000 rpm for 2 min to collect the eluate. Afterwards, the pH of the sample was neutralized with Tris-HCl (pH 8.0) buffer. Samples were mixed with LDS loading buffer and heated at 70°C for 10 min and subjected to immunoblot analysis using α-WRNIP1, α-FANCD2 or α-His antibodies.

Ubiquitin binding assay

Flag-HA-WRNIP1 purified from Sf9 cells was used for ubiquitin binding assays. Protein purification from Sf9 cells were carried out as previously described (Liang et al., 2016). 2 μg Flag-HA-WRNIP1 or Flag-HA-WRNIP1-19R were mixed with 2 μg His-ubiquitin in a binding buffer (300 mM KCl, 40 mM Tris-HCl (pH 8.0), 100 μg/ml BSA and 4 mM β-mercaptoethanol) and incubated at 30°C for 1 h. Then the protein mixture was incubated with α-HA Sepharose beads at 4°C for 30 min. After incubation the beads were transferred to an empty chromatography column where beads were washed by a wash buffer (300 mM KCl, 100 mM Tris-HCl (pH 8.0), 0.2 mM PMSF, 2 mM β-mercaptoethanol and 0.1% Tween-20 (v/v)). The wash buffer was removed by spinning at 1,000 rpm for 10 s before proteins were eluted by 1x LDS loading buffer. Eluted proteins were examined by 9% and 20% SDS-PAGE and immunoblot analysis using antibodies against WRNIP1 and Ubiquitin, respectively.

Live-cell microscopy

Cells used for live-cell microscopy were plated into microscopy dishes **(**MatTek Glass Bottom Microwell Dishes P35G-1.5-20-C) and pre-treated accordingly (in case of WRNIP1-EGFP cells and the variants of WRNIP1-EGFP with point mutations, the media was supplemented with 0.005 μg/ml of Doxycycline 3 h before imaging). The media was supplemented with 500 ng/ml of TMP (Sigma, T6137) and the dish was placed in the stand of the PE UltraVIEW spinning disk microscope for 15 min. The used objective properties were as follows: magnification: 60x, numerical aperture: 1.4, immersion medium: oil (N = 1.514). 405 nm wavelength laser irradiation was used to irradiate the samples along indicated lines and the before and after images were acquired for each sample. The images were acquired in the green (488 nm) and/or red (561 nm) fluorescence channels. The camera used for acquisition was Hamamatsu C9100-13. All images were acquired and processed using the Volocity software. After acquisition the images were analyzed with FiJi software where the following measurements were taken. First, the areas where the cells were irradiated were circled by hand using the free-select tool. It was necessary to do this by hand for each image because the irradiated areas sometimes move slightly during long acquisitions due to cell migration. Second, measurement of the brightness of the selected field was performed and recorded. Third, the rest of the cell nucleus was selected avoiding the irradiated area, and the same measurement was performed. Fourth, the background was selected and its intensity measured. Then for each cell the following formula was used: $Sx = (Ax-B)/(Cx-B)$, where Ax represents the brightness of the stripe, Cx the brightness of the rest of the rest of the cell nucleus and B the brightness of the background in given field of view. The formula is returning Sx, which represents the relative brightness of the stripe compared to the rest of the nucleus in the given cell. After obtaining Sx values for each of the cells in the view field and for each time point of interest, the average S value was calculated along with the standard error (here standard deviation divided by the square root of the number of all the cells taken into consideration in the given view field). To make the comparison between the experiments easier, measurements were then normalized in such way that the first time point (before the irradiation) would always equal 1. To normalize, the difference between the first time point and 1 was added or subtracted in all later measurements (so for example if the average value for all cells in the first time point was giving the value of 1.12, 0.12 would be subtracted from this and all future time points). It was a necessary adjustment since, the distribution of the proteins used in our experiments is not uniform in the nucleus, with dark areas and sometimes bright foci. Since we usually tried to locate the irradiation area in such a way to avoid irradiating these non-uniform areas, its brightness would not exactly be equal to that of the rest of the nucleus even in the first time point before the irradiation.

Analysis of nuclear FANCD2 foci using live-cell imaging was performed using cells expressing Halo-tagged FANCD2, either with or without additional WRNIP1 knockdown using shRNA. Immediately before the experiment, the cells were labeled with Janelia Fluor 549 for 30 min and then washed thrice in DMEM supplemented with 10% FBS and 2 mM L-Gln (without Phenol Red). They were then left to recover for 30 min in the incubator. The cells were subsequently exposed to 160 ng/mL mitomycin C (MMC) and followed for 4 h. Images from twelve fields of view, with 21 z stacks each, were collected at 0 h, 2 h, and 4 h time points. For each time point, the percentage of cells with > 10 foci/nucleus was quantified. For control cells, a total of 373, 414 and 361 cells were quantified from 3 independent experiments at 0 h, 2 h, and 4 h time points, respectively. For WRNIP1 knockdown cells, a total of 465, 588 and 511 cells were quantified from 3 independent experiments at 0 h, 2 h, and 4 h time points, respectively. Error bar shows mean ± SEM from 3 independent experiments. Statistical significance was determined using the Student's t test with a p value cut-off of 0.05.

QUANTIFICATION AND STATISTICAL ANALYSIS

Statistical parameters, including statistical tests used, number of events quantified, standard error of the mean, and statistical significance are reported in the figures and in the figure legends. Statistical analysis has been performed using Microsoft Office Excel software and statistical significance is determined by the value of $p < 0.05$.

Cell Reports, Volume *32*

Supplemental Information

WRNIP1 Is Recruited to DNA Interstrand Crosslinks and Promotes Repair

Anna Socha, Di Yang, Alicja Bulsiewicz, Kelvin Yaprianto, Marian Kupculak, Chih-Chao Liang, Andreas Hadjicharalambous, Ronghu Wu, Steven P. Gygi, and Martin A. Cohn

Figure S1

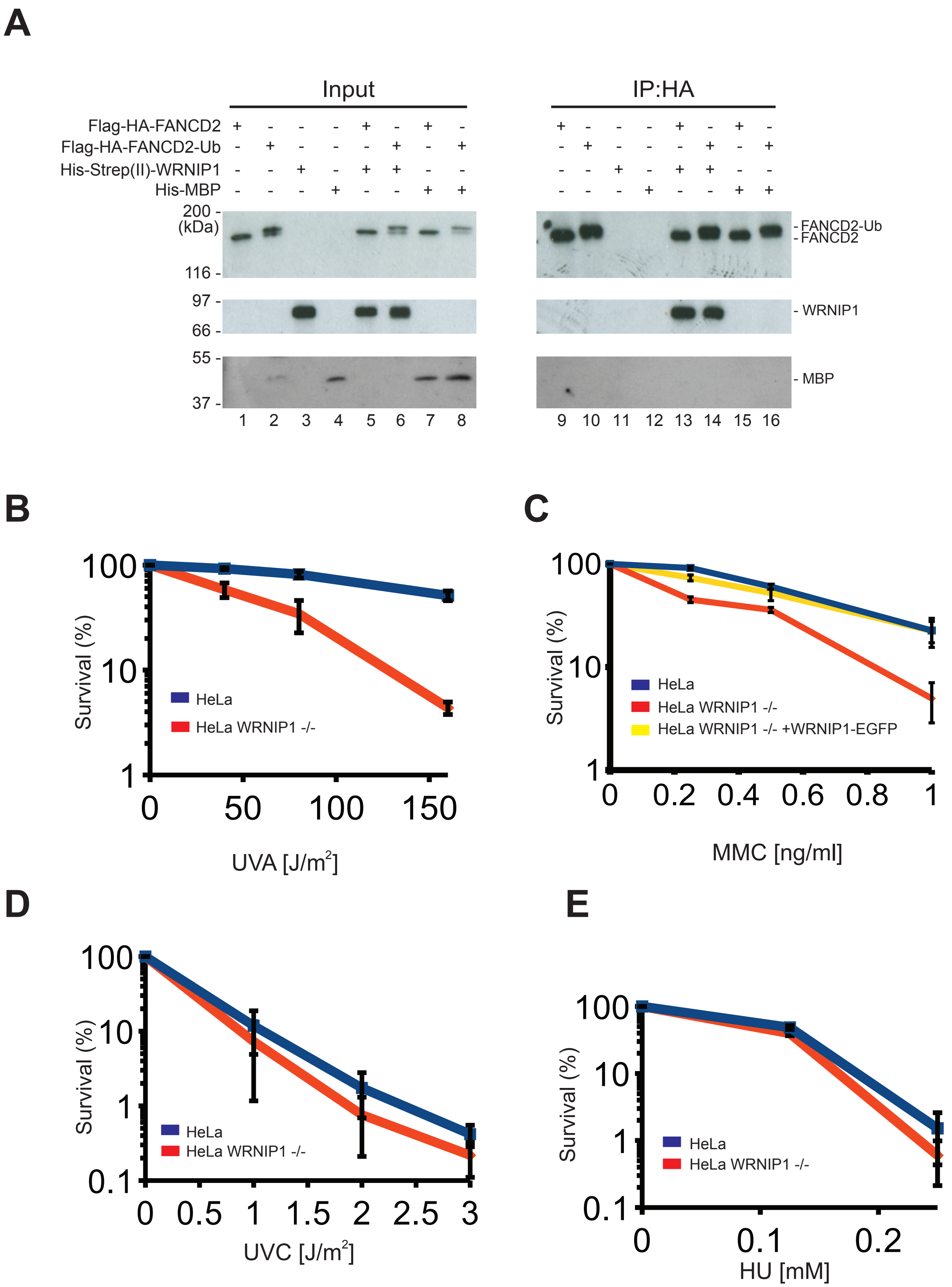

Figure S1. WRNIP1 is important for ICL repair. Relating to Figures 1 and 2. A) Immunoblot analysis of *in vitro* protein-protein interaction assay showing a direct interaction between WRNIP1 and FANCD2. B) The CRISPR/Cas9 technique was used to disrupt the *WRNIP1* gene in HeLa cells, creating a WRNIP1 deficient HeLa cell line. Clonogenic survival assay of HeLa and HeLa WRNIP1-/- cells in response to TMP/UVA (mean ± SEM, n=2). C) Clonogenic survival assay of HeLa and HeLa WRNIP1-/- cells in response to MMC (mean ± SEM, n=2). D) Clonogenic survival assay of HeLa and HeLa WRNIP1-/- cells in response to UVC (mean ± SEM, n=2). E) Clonogenic survival assay of HeLa and HeLa WRNIP1-/- cells in response to HU (mean ± SEM, n=2).

Figure S2

A

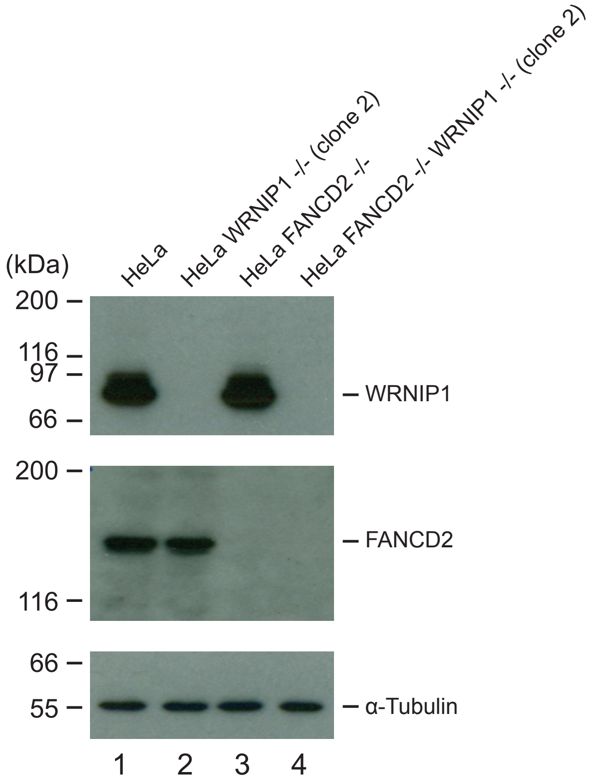

B

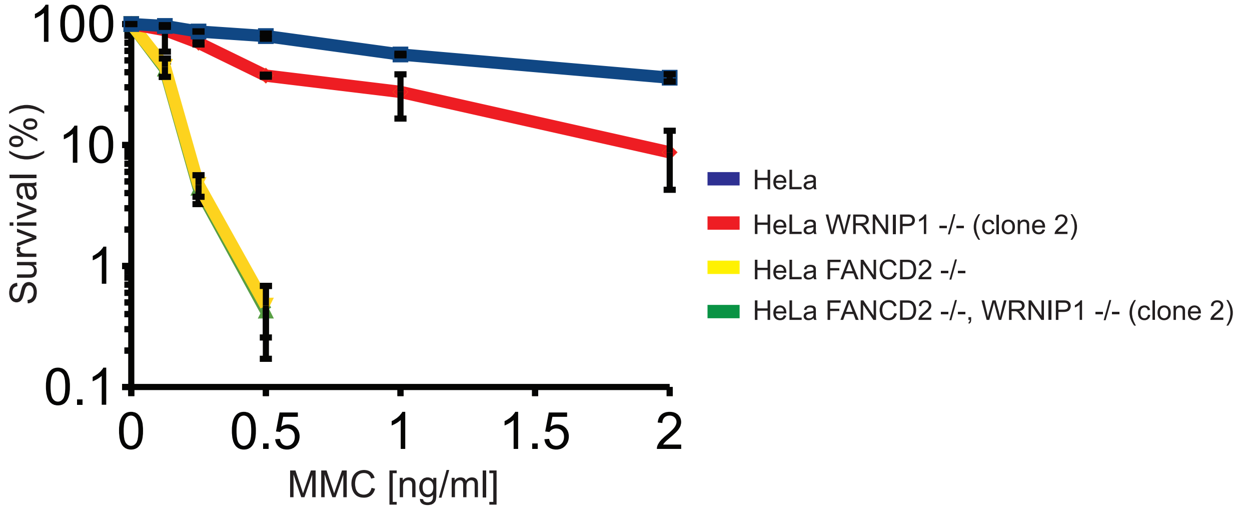

Figure S2. WRNIP1 is important for ICL repair. Relating to Figure 2. A) The CRISPR/Cas9 technique was used to disrupt the *WRNIP1* and *FANCD2* genes in HeLa cells, creating HeLa WRNIP -/- (clone 2), HeLa FANCD2 -/- and HeLa WRNIP -/- FANCD2 -/- (clone 2) cell lines. Immunoblot analysis. B) Clonogenic survival assay of the indicated cell lines in response to MMC. Mean ± SEM, n=2.

Figure S3

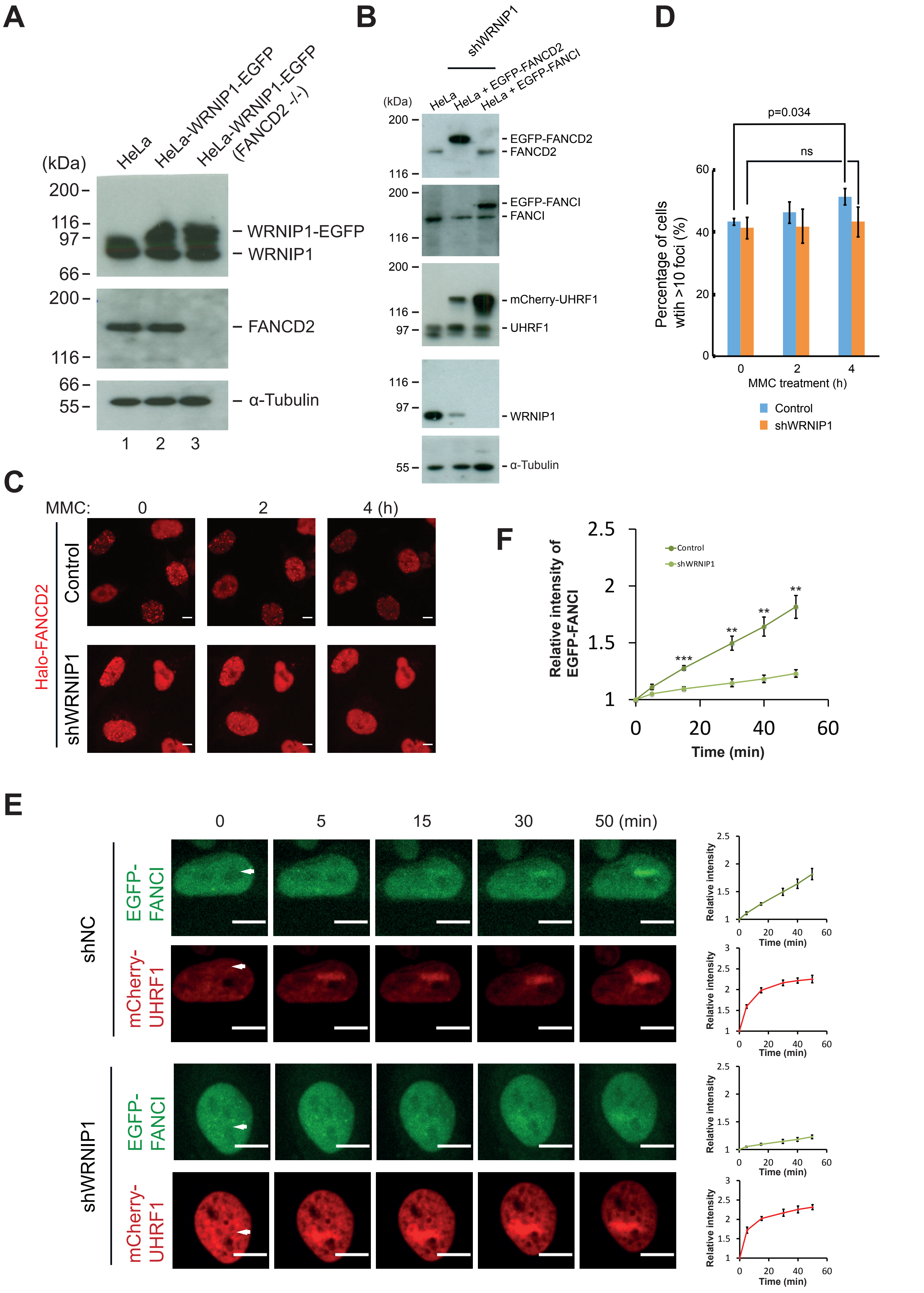

(legend on next page)

Figure S3. WRNIP1 is recruited to ICLs. Relating to Figure 3. A) Immunoblot analysis showing expression of WRNIP1-EGFP in Flp-in T-REx HeLa and Flp-in T-REx HeLa FANCD2 -/- cells. B) Immunoblot analysis showing expression of EGPF-FANCD2, EGPF-FANCI and mCherry-UHRF1 in HeLa cells expressing either a control shRNA encoding a scramble sequence, or, an shRNA targeting WRNIP1. C) Live-cell imaging of HeLa-Halo-FANCD2 cells and HeLa-Halo-FANCD2 cells where WRNIP1 was depleted using shRNA. Cells were treated with 160 ng/ml MMC and followed over time. Scale bar, 10 µm. D) Quantification of (C). A total of 373, 414 and 361 cells were quantified for control cells at 0, 2, and 4h time points, respectively. A total of 465, 588 and 511 cells were quantified for cells where WRNIP1 was depleted at 0, 2, and 4h time points, respectively. Mean ± SEM, ns, not significant. E) Live-cell imaging of HeLa-EGFP-FANCI, mCherry-UHRF1 cells. Cells were treated with TMP and microirradiated at the indicated areas (white arrows) and followed over time. Quantification showing relative intensities of the irradiated areas. Mean ± SEM. A total of 5 cells were quantified for each chart. Scale bar, 10 µm. F) Combination of quantification data from (E), showing recruitment of EGFP-FANCI in control cells and in cells where WRNIP1 is depleted. ** indicates that $p < 0.01$, *** indicates that $p < 0.001$. Mean ± SEM.

Figure S4

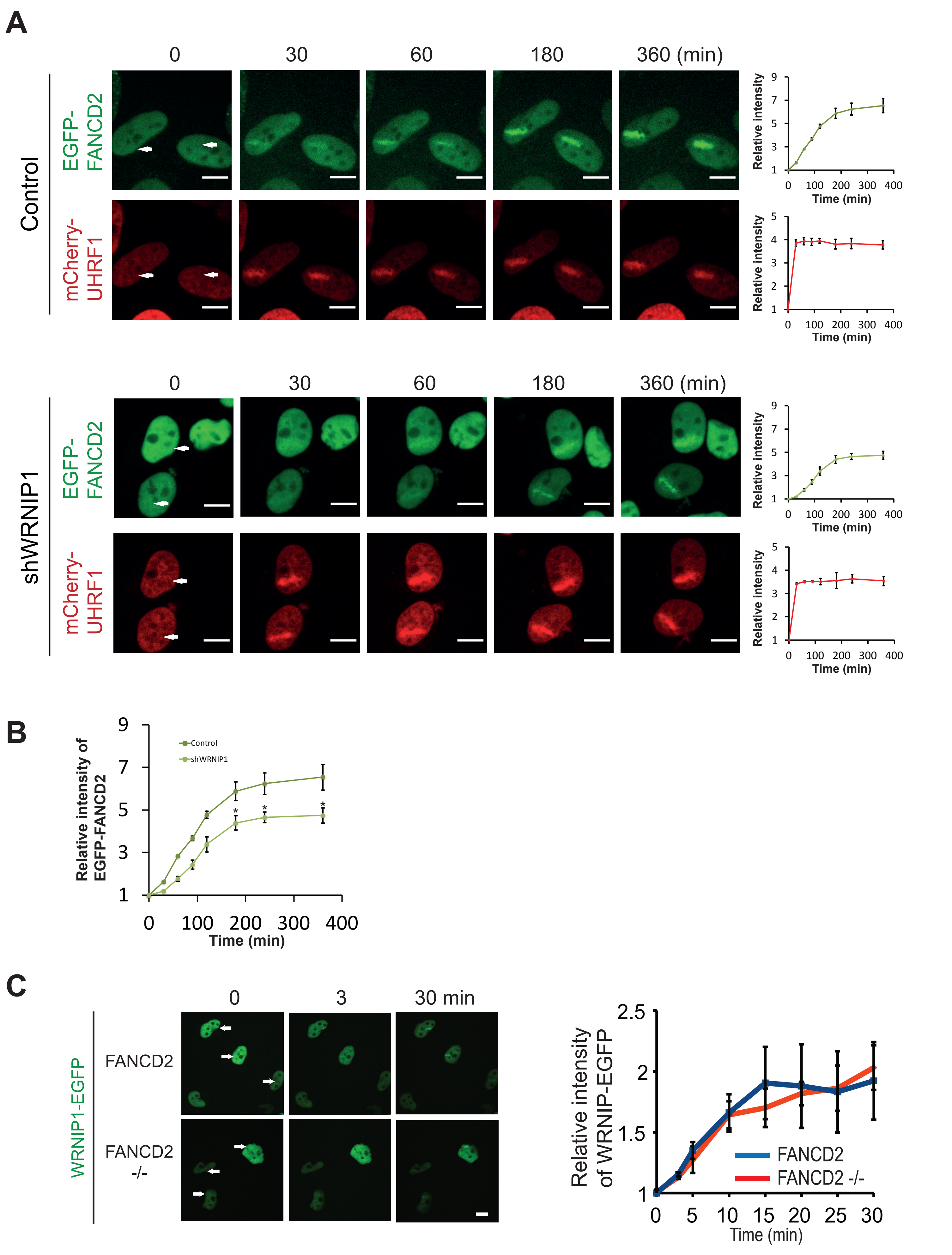

(legend on next page)

Figure S4. WRNIP1 is recruited to ICLs. Relating to Figure 3. A) Live-cell imaging of HeLa-EGFP-FANCD2, mCherry-UHRF1 cells. Cells were treated with TMP and microirradiated at the indicated areas (white arrows) and followed over time. Quantification showing relative intensities of the irradiated areas. A total of 4 cells were quantified for each chart. Mean ± SEM. Scale bar, 10 µm. B) Combination of quantification data from (A), showing recruitment of EGFP-FANCD2 in control cells and in cells where WRNIP1 is depleted. Mean ± SEM. * indicates that $p < 0.05$. C) Live-cell imaging of Flp-in T-Rex HeLa-WRNIP1-EGFP and Flp-in T-Rex HeLa FANCD2 -/- +WRNIP1-EGFP cells. Cells were treated with Doxycycline and TMP and microirradiated at the indicated areas (white arrows) and followed over time. Scale bar, 10 µm. Quantification showing relative intensities of the irradiated areas. Data represent 2 independent experiments (n=2) and 14 cells in total were quantified, 9 control cells and 5 FANCD2 deficient cells. Mean ± SEM.

Figure S5

A

Sites	Frequency
K62	2
K70	5
K141	2
K287	2
K301	2
K310	2
K464	2
K482	1
K627	1
K661	1

B

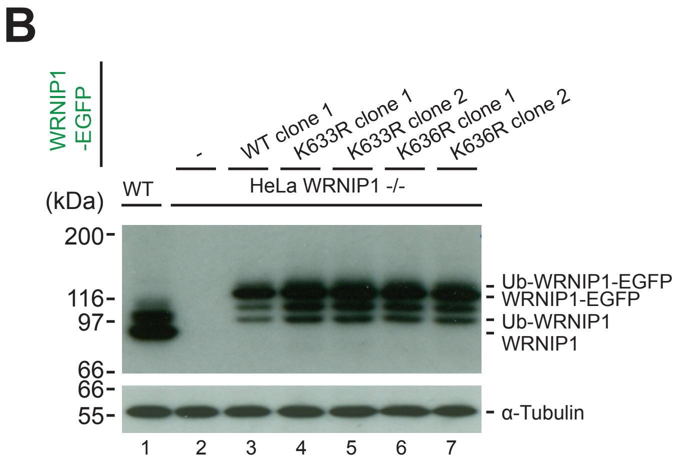

C

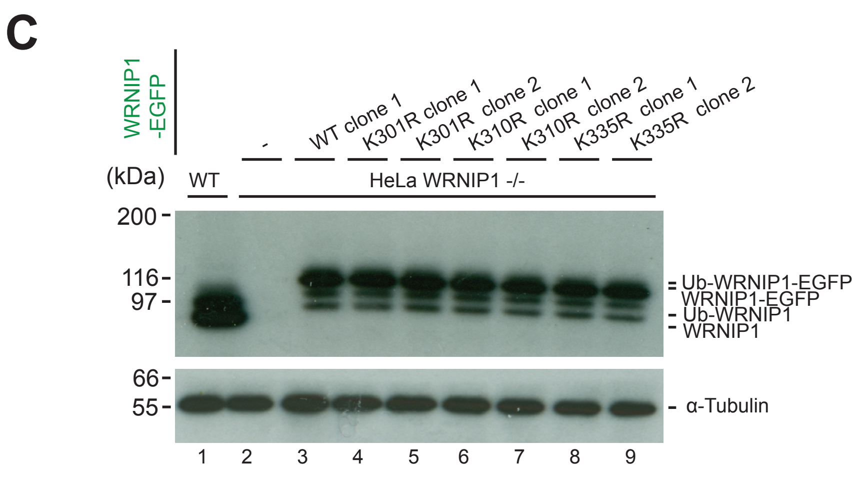

D

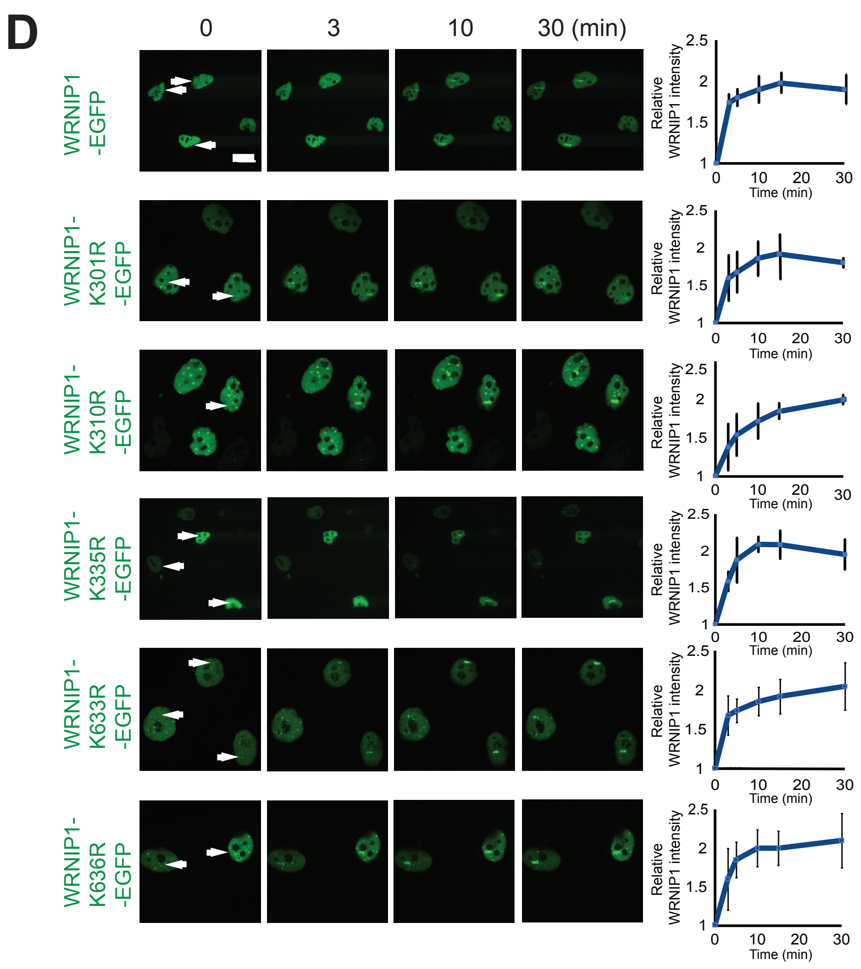

E

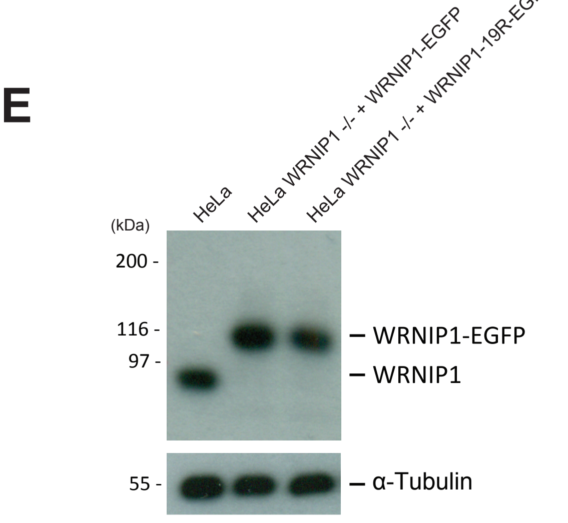

F

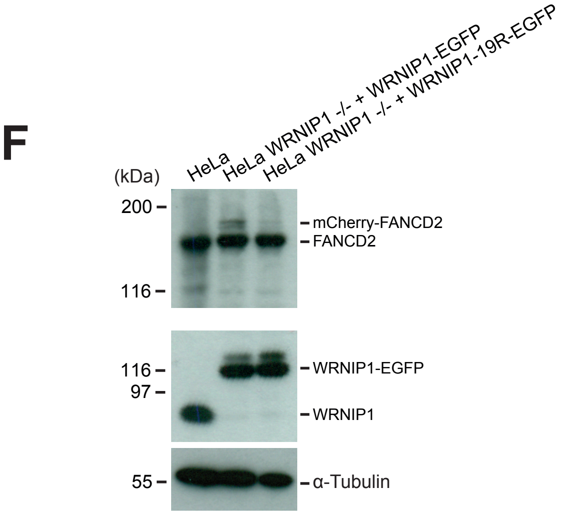

G

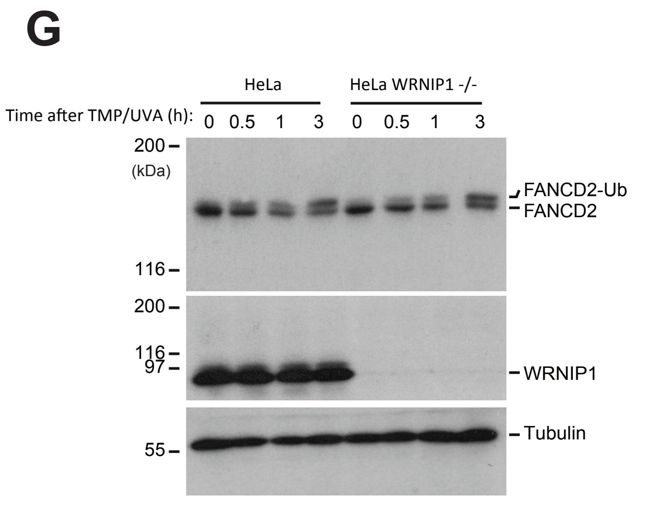

(legend on next page)

Figure S5. WRNIP1 is ubiquitinated. Relating to Figure 4. A) Details of Gly-Gly-containing peptides identified from WRNIP1. B-C) The CRISPR/Cas9 technique was used to disrupt the *WRNIP1* gene in HeLa cells. The indicated derivatives of WRNIP1-EGFP were then expressed in the WRNIP1 deficient cell line. Immunoblot analysis. D) Live-cell imaging of Flp-in T-Rex HeLa WRNIP1 -/- cells complemented with the indicated derivatives of WRNIP1-EGFP. Cells were treated with Doxycycline and TMP and microirradiated at the indicated areas (white arrows) and followed over time. Quantification showing relative intensities of the irradiated areas. Data represent 2 independent experiments (n=2) and the following number of cells were analysed: 7 cells for K301, 6 cells for K310R, 6 cells for K335R, 6 cells for K633R and 5 cells for K636R. Mean ± SEM. Scale bar, 10 µm. E) Immunoblot analysis showing expression of WRNIP1-EGFP and WRNIP1-19R-EGFP in HeLa WRNIP1 -/- cells. F) Immunoblot analysis showing expression of WRNIP1-EGFP or WRNIP1-19R-EGFP, and mCherry-FANCD2 stably expressed in HeLa WRNIP1 -/- cells. G) Immunoblot analysis of FANCD2 and WRNIP1 in HeLa and HeLa WRNIP1 -/- cells following treatment with TMP/UVA.

Figure S6

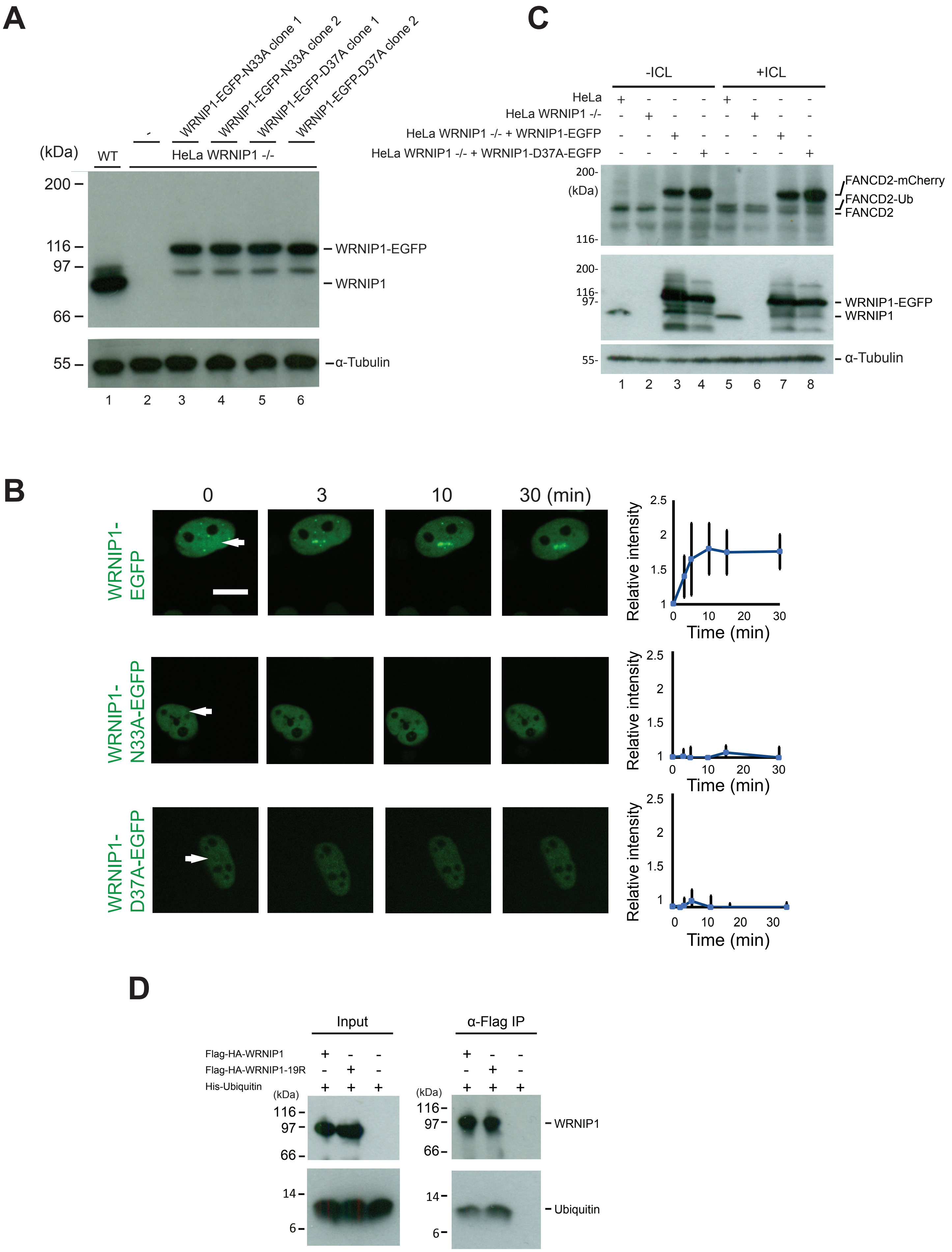

(legend on next page)

Figure S6. The UBZ domain of WRNIP1 is necessary for its ICL repair activity. Relating to Figure 6. A) The CRISPR/Cas9 technique was used to disrupt the WRNIP1 gene in HeLa Flp-in T-Rex cells. The resulting Flp-in T-Rex HeLa-WRNIP1 -/- cells were then complemented with the indicated derivatives of WRNIP1-EGFP. Cells were treated with Doxycycline. Immunoblot analysis using the indicated antibodies. B) Live-cell imaging of Flp-in T-Rex HeLa-WRNIP1-EGFP, HeLa-WRNIP1-N22A-EGFP and HeLa-WRNIP1-D37A-EGFP cells. Cells were treated with Doxycycline and TMP and microirradiated at the indicated areas (white arrows) and followed over time. Quantification showing relative intensities of the irradiated areas. Data represent 2 independent experiments (n=2) and 5 cells in total were quantified for WRNIP1-EGFP, 9 cells in total were quantified for WRNIP1-N33A-EGFP and 5 cells in total were quantified for WRNIP1-D37A-EGFP. Mean ± SEM. Scale bar, 10 μm. C) Immunoblot analysis showing stable expression of mCherry-FANCD2, WRNIP1-EGFP or WRNIP1-D37A-EGFP in HeLa WRNIP1 -/- cells in untreated cells, or in cells treated with TMP/UVA 3h prior to harvest to introduce ICLs. D) In vitro protein-protein interaction assay showing a direct interaction between WRNIP1 and Ubiquitin, and WRNIP1-19R and Ubiquitin. Immunoblot analysis using anti-WRNIP1 and anti-Ubiquitin antibodies as indicated.

Figure S7

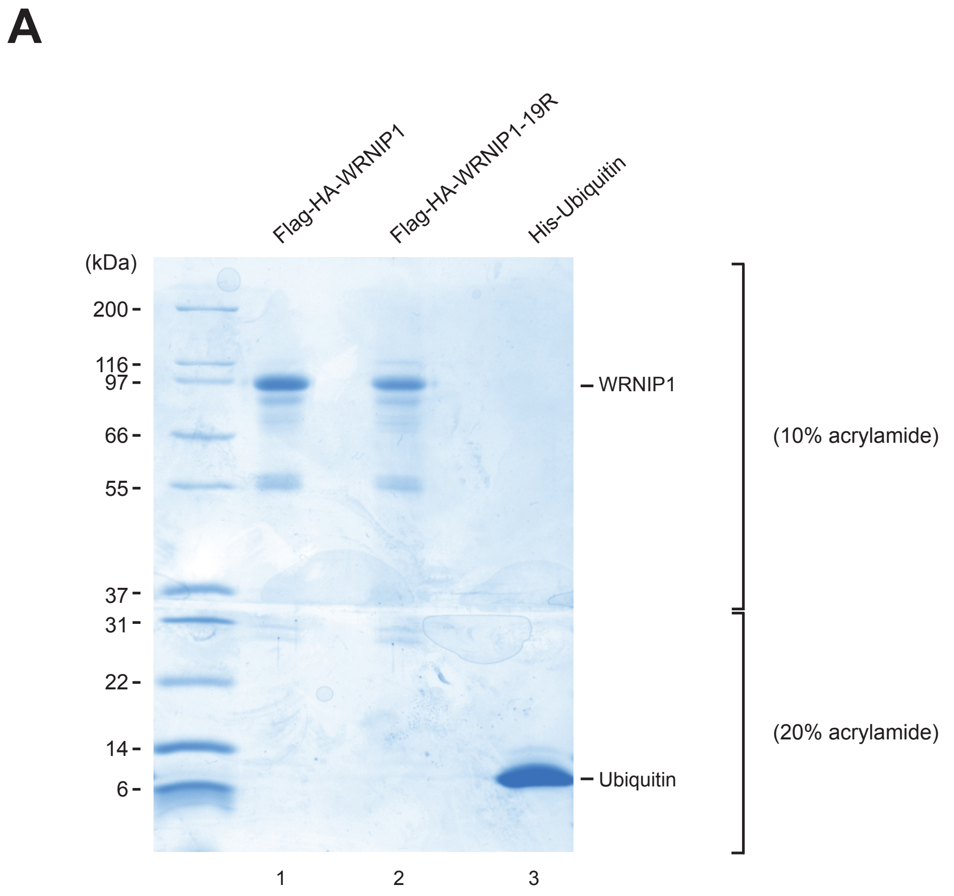

Figure S7. The UBZ domain of WRNIP1 is necessary for its ICL repair activity. Relating to Figure 6. A) Coomassie blue staining of the recombinant proteins used in the *in vitro* protein-protein interaction assay shown in Figure 6E. The upper part of the separation gel contained 10% acrylamide and lower part contained 20% acrylamide.
